# Role of the renal sympathetic nerve in renal glucose metabolism during the development of type 2 diabetes in rats

**DOI:** 10.1007/s00125-015-3771-9

**Published:** 2015-10-08

**Authors:** Kazi Rafiq, Yoshihide Fujisawa, Shamshad J. Sherajee, Asadur Rahman, Abu Sufiun, Hiroyuki Kobori, Hermann Koepsell, Masaki Mogi, Masatsugu Horiuchi, Akira Nishiyama

**Affiliations:** Department of Pharmacology, Faculty of Medicine, Kagawa University, 1750-1 Ikenobe, Miki-cho, Kita-gun, Kagawa, 761-0793 Japan; Life Science Research Center, Faculty of Medicine, Kagawa University, Kagawa, Japan; Department of Molecular Plant Physiology and Biophysics, University of Würzburg, Julius-von-Sachs-Institute, Julius-von-Sachs-Platz 2, 97082 Würzburg, Germany; Department of Molecular Cardiovascular Biology and Pharmacology, Graduate School of Medicine, Ehime University, Matsuyama, Japan

**Keywords:** Blood pressure, Glucose metabolism, Insulin resistance, Renal sympathetic denervation (RDX), *Sglt2*, *Slc5a2*, Sodium-glucose cotransporter 2, Type 2 diabetes

## Abstract

**Aims/hypothesis:**

Recent clinical studies have shown that renal sympathetic denervation (RDX) improves glucose metabolism in patients with resistant hypertension. We aimed to elucidate the potential contribution of the renal sympathetic nervous system to glucose metabolism during the development of type 2 diabetes.

**Methods:**

Uninephrectomised diabetic Otsuka Long-Evans Tokushima Fatty (OLETF) rats underwent RDX at 25 weeks of age and were followed up to 46 weeks of age.

**Results:**

RDX decreased plasma and renal tissue noradrenaline (norepinephrine) levels and BP. RDX also improved glucose metabolism and insulin sensitivity, which was associated with increased in vivo glucose uptake by peripheral tissues. Furthermore, RDX suppressed overexpression of sodium-glucose cotransporter 2 (*Sglt2* [also known as *Slc5a2*]) in renal tissues, which was followed by an augmentation of glycosuria in type 2 diabetic OLETF rats. Similar improvements in glucose metabolism after RDX were observed in young OLETF rats at the prediabetic stage (21 weeks of age) without changing BP.

**Conclusions/interpretation:**

Here, we propose the new concept of a connection between renal glucose metabolism and the renal sympathetic nervous system during the development of type 2 diabetes. Our data demonstrate that RDX exerts beneficial effects on glucose metabolism by an increase in tissue glucose uptake and glycosuria induced by *Sglt2* suppression. These data have provided a new insight not only into the treatment of hypertensive type 2 diabetic patients, but also the pathophysiology of insulin resistance manifested by sympathetic hyperactivity.

**Electronic supplementary material:**

The online version of this article (doi:10.1007/s00125-015-3771-9) contains peer-reviewed but unedited supplementary material, which is available to authorised users.

## Introduction

Insulin resistance is a common feature of the metabolic syndrome and type 2 diabetes, which is often associated with sympathetic nervous system hyperactivity [1–3]. A potential bidirectional relationship between sympathetic hyperactivity and insulin resistance, hypertension, diabetes or cardiovascular injury has also been suggested [4, 5]. Renal afferent and efferent nerves mediate a sympathetic signal between the kidney and central sympathetic nervous system, respectively [6, 7]. Obese and diabetic patients show an increase in renal sympathetic nerve activity, which is associated with hypertension, insulin resistance and cardio-renal syndrome [6, 8, 9]. In patients with heart failure, increases in noradrenaline (norepinephrine [NE]) overflow from the heart and the kidney were also observed [10].

Accumulating clinical evidence has indicated the effectiveness of catheter-based renal sympathetic denervation (RDX) in reducing BP [11–13] and left ventricular hypertrophy [14]. We have demonstrated that RDX suppresses the onset of albuminuria in cardiac volume overload rats [15], highlighting the importance of the renal sympathetic nervous system in cardio-renal syndrome. SYMPLICITY HTN-3, a prospective, single-blind, randomised clinical trial did not show a significant systolic BP (SBP) reduction in patients with resistant hypertension 6 months after renal-artery denervation [16]. However, Mahfoud et al [17] have shown that RDX improves glucose metabolism, insulin sensitivity and BP control in patients with resistant hypertension in the absence of any changes in body weight or lifestyle. Furthermore, Witkowski et al [18] have shown that RDX improves comorbid refractory hypertension, glucose intolerance and obstructive sleep apnoea in patients with resistant hypertension. More interestingly, it has also been demonstrated that RDX exerts beneficial effects not only on BP control but also on insulin sensitivity in polycystic ovary syndrome patients [19]. However, the mechanism responsible for the beneficial effect of RDX on glucose metabolism has not yet been clarified.

In the present study, we aimed to determine the precise mechanism by which the renal sympathetic nervous system contributes to glucose metabolism during the development of type 2 diabetes. Here, we demonstrated that RDX markedly improved insulin sensitivity and glucose metabolism by increasing glucose uptake in peripheral tissues of obese type 2 diabetic Otsuka Long-Evans Tokushima Fatty (OLETF) rats. Interestingly, improvement in glucose metabolism was also associated with suppression of sodium-glucose cotransporter 2 (*Sglt2* [also known as *Slc5a2*]) overexpression in the kidney, leading to enhanced glycosuria. Similar data were observed in young obese OLETF rats at the prediabetic stage. These data suggest the novel concept of a connection between renal glucose metabolism and the renal sympathetic nervous system, which offers new insight into the management of hypertensive patients with obesity, the metabolic syndrome and type 2 diabetes.

## Methods

### Animals

Experimental protocols and animal care were performed according to the guidelines for the care and use of animals established by Kagawa University, Japan. Four-week-old male obese OLETF and control lean Long-Evans Tokushima Otsuka (LETO) rats (Hoshino Laboratory, Bando, Japan) were maintained under a controlled temperature (24 ± 2°C) and humidity (55 ± 5%), with a 12 h light/dark cycle. Previous studies have shown that OLETF rats exhibit the prediabetic metabolic syndrome phase from 10 to 20 weeks of age, and the type 2 diabetic phase from 25 to 30 weeks of age [20, 21]. Thus, in this study, experiments of protocols 1 and 2 were conducted in OLETF rats at the diabetic stage (from 25 to 46 weeks of age) and the prediabetic stage (4 to 21 weeks of age).

### Experimental protocols

#### Protocol 1

All rats were subjected to right uninephrectomy under isoflurane anaesthesia at 5 weeks of age. Then, left kidney RDX was performed at 25 weeks of age and rats were grouped as follows: uninephrectomised control lean LETO rats (LETO, *n* = 10), uninephrectomised-denervated LETO rats (LETO+RDX, *n* = 10), uninephrectomised OLETF rats (OLETF, *n* = 10), uninephrectomised-denervated OLETF rats (OLETF+RDX, *n* = 10). In protocol 1, OGTT was performed at 30, 35 and 45 weeks of age (5, 10 and 20 weeks after RDX, respectively). Twenty-four hour urine samples were collected to determine urinary NE, glucose and protein excretion. Fasting blood glucose and body weight were measured at 25, 30, 35, 40 and 45 weeks of age.

#### Protocol 2

The rats were subjected to right uninephrectomy at 5 weeks of age. Left kidney RDX was performed at 6 weeks of age and rats were grouped as follows: LETO (*n* = 8), LETO+RDX (*n* = 8), OLETF (*n* = 8), OLETF+RDX (*n* = 8). In both protocols 1 and 2, right uninephrectomy was performed to prevent reno-renal reflexes from the right kidney, as previously described [22, 23]. In protocol 1, OGTT was performed at 14 and 20 weeks of age (8 and 14 weeks after RDX, respectively). Twenty-four hour urine samples were collected at 13, 17 and 20 weeks of age. Fasting blood glucose and body weight were measured at 5, 9, 14, 18 and 20 weeks of age.

### RDX

**Fig. 1:**
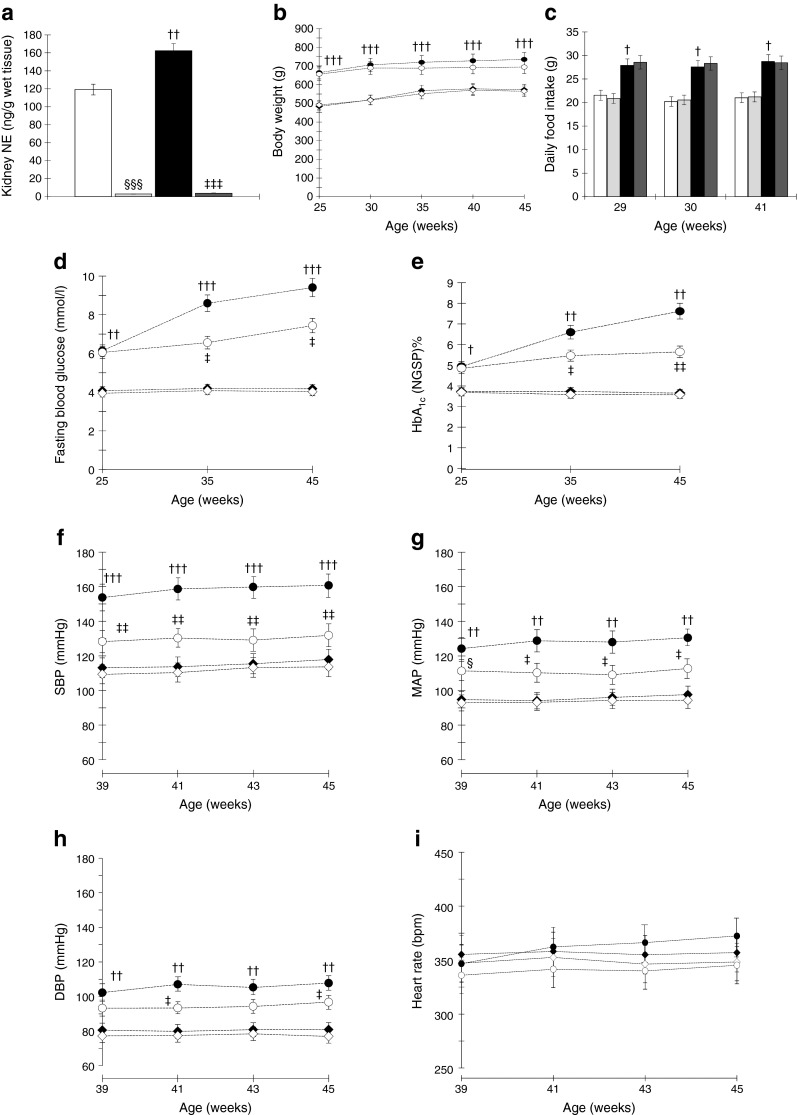
(**a**) OLETF rats show significantly higher kidney tissue NE levels compared with LETO rats. Kidney tissue NE levels in OLETF rats subjected to RDX are almost undetectable. (**b**, **c**) OLETF rats show higher body weight (**b**) and daily average food intake (**c**) compared with LETO rats, which were not affected by RDX. (**d**, **e**) OLETF rats show markedly elevated fasting blood glucose (**d**) and HbA_1c_ (**e**) levels compared with LETO rats, which are suppressed by RDX. (To convert values for HbA_1c_ in DCCT % into mmol/mol, subtract 2.15 and multiply by 10.929.) (**f**–**h**) OLETF rats show markedly increased SBP (**f**), MAP (**g**) and DBP (**h**) compared with LETO rats. RDX suppresses SBP, MAP and DBP in OLETF rats. However, heart rate was not different among the groups (**i**). ^†^ *p* < 0.05, ^††^ *p* < 0.01, ^†††^ *p* < 0.005 LETO vs OLETF; ^‡^ *p* < 0.05, ^‡‡^ *p* < 0.01, ^‡‡‡^ *p* < 0.005 OLETF vs OLETF+RDX; ^§^ *p* < 0.05 LETO vs LETO+RDX; ^§§§^ *p* < 0.005 LETO vs LETO+RDX. White bars and white diamonds, LETO group; black bars and black circles, OLETF group; light grey bars and black diamonds, LETO+RDX; dark grey bars and white circles, OLETF+RDX

Uninephrectomised rats were subjected to RDX under isoflurane anaesthesia. Complete RDX was achieved by carefully cutting and stripping all of the visible renal nerves along the renal artery and vein from the aorta to the hilum of the kidney, and painting these vessels with a solution of 10% phenol in ethanol (vol./vol.) [15, 24]. This method ablates the afferent and efferent renal nerves [24, 25]. At the end of each experiment, renal tissue was harvested and NE content was measured to confirm the completeness of RDX [24, 25]. In both protocols 1 and 2, the kidney NE content in all renal denervated rats was almost undetectable (<5 ng/g tissue) (Fig. 1a and Electronic Supplementary Material [ESM] Fig. 1a).

### OGTT and hyperinsulinaemic–euglycaemic clamp study

OGTT and hyperinsulinaemic–euglycaemic clamp studies were performed as described previously [26–29]. Details are provided in the ESM Methods.

### Telemetric sensor implantation

BP profile (mean arterial pressure [MAP], SBP and diastolic BP [DBP]) and heart rate were assessed by radio-telemetry system (Data Sciences International, St Paul, MN, USA) in conscious rats, as described previously [30, 31] in a separate set of animals (*n* = 5 for each treatment group). Details are provided in the ESM Methods.

### Measurement of rate constant of net tissue uptake of 2-[^3^H]deoxy-d-glucose

In vivo uptake of 2-[^3^H]deoxy-d-glucose (2-[^3^H]DG) in peripheral tissues (Brown adipose tissue [BAT], retroperitoneal white adipose tissues [WAT], skeletal muscles [soleus muscles] and liver) was measured as described previously [32, 33] in another group of animals (*n* = 6 for each treatment group). The rate constant of net tissue uptake of 2-[^3^H]DG was calculated, as described previously [34]. Details are provided in the ESM Methods.

### Sample collection

At the end of each observation period, blood was collected and kidneys perfused with an isotonic saline under anaesthesia with sodium pentobarbital (65 mg/kg, i.p.). Details are provided in the ESM Methods.

### Histological examination

Kidney tissues were fixed with 10% paraformaldehyde (wt/vol.), embedded in paraffin, sectioned into 4 μm thick slices and stained with periodic acid-Schiff (PAS) [35, 36]. The percentage of PAS-positive areas was measured using image analysis software, WinROOF (Mitani Corporation, Tokyo, Japan). A total of 25–30 glomeruli were examined per rat and the average percentage of affected lesions was calculated [35, 36].

### Real-time RT-PCR

The mRNA expression in renal cortical tissues was analysed by RT-PCR using a LightCycler FastStart DNA Master SYBR Green I kit and an ABI Prism 7,000 Sequence Detection System (Applied Biosystems, Foster City, USA) as previously described [15, 35]. The oligonucleotide primer sequences for rat and human are listed in ESM Tables 1 and 2, respectively. All data from in vivo studies are expressed as the relative difference in expression compared with LETO rats after normalisation for β-actin expression. Data from in vitro studies are expressed as the relative difference in expression compared with 5 mmol/l glucose after normalisation for β-actin expression.

### Immunohistochemistry for sodium-glucose cotransporter 2

Renal tissue fixation and immunofluorescence study for Sodium-glucose cotransporter 2 (SGLT2) were performed as described previously [37]. Details are provided in the ESM Methods.

### Other analytical procedures

Details are provided in the ESM Methods.

### Cell culture experiments

HK2 cells (immortalised human kidney proximal tubule epithelial cells) were cultured in DMEM supplemented with 10% FBS [15]. After reaching 40% confluence in a six-well plate, cells were serum deprived for 24 h before experimental manipulation. All experiments were performed under serum-free conditions and the cells remained viable in this condition in a nonproliferating state. Quiescent cells were then treated with normal glucose medium (5 mmol/l) as well as high glucose (15 mmol/l) medium with or without 100 nmol/l NE for 12 and 24 h. Details are provided in the ESM Methods.

### Statistical analyses

The data are expressed as means ± SEM. Statistical comparisons of differences among groups were performed using one-way repeated-measures ANOVA, followed by the Newman–Keuls post hoc test. SBP, DBP, urinary protein excretion, urinary glucose excretion, urinary NE excretion, fasting blood glucose, HbA_1c_ and body weight were compared using two-way repeated-measures ANOVA followed by the same post hoc test. Values of *p* < 0.05 were considered statistically significant. Data and statistical analyses were performed using GraphPad Prism version 5 for Windows (Graph Pad Software, San Diego, CA, USA).

## Results

### General variables and BP profiles

In both protocols 1 and 2, OLETF rats showed elevated renal cortical tissue NE levels compared with age-matched LETO rats. Kidney NE levels in all RDX rats were almost undetectable, indicating that RDX was successfully completed (Fig. 1a, ESM Fig. 1a). Renal cortical tissue adrenaline levels are elevated in OLETF rats in protocol 1 and were unaffected by RDX in both protocols 1 and 2 (Table 1). During the experimental period, OLETF rats had higher body weight gain and daily food intake than LETO rats. RDX at diabetic or prediabetic stages did not affect daily food intake and body weight gain either in LETO or OLETF rats (Fig. 1b,c, ESM Fig. 1b,c). In both protocols 1 and 2, fasting blood glucose and HbA_1c_ were significantly higher in OLETF than LETO rats, and were significantly decreased by RDX (Fig. 1d,e, ESM Fig. 1d,e). At 45 weeks of age in protocol 1, the plasma insulin level was significantly higher in OLETF than LETO rats (1.03 ± 0.13 and 33.46 ± 2.7 pmol/l, *p* < 0.0001, respectively), and was attenuated by RDX in OLETF rats (20.90 ± 6.1 pmol/l, *p* < 0.05). Both diabetic and prediabetic stage OLETF rats have significantly higher plasma triacylglycerol, total cholesterol and NEFA, which were suppressed by RDX (Tables 2 and 3). Plasma sodium and potassium levels were not altered by RDX (Tables 2 and 3).

**Table 1:** Kidney tissues adrenaline levels at the diabetic stage (protocol 1) and at the prediabetic stage (protocol 2)

	Diabetic stage (at 46 weeks of age); adrenaline (ng/g wet tissue)	Prediabetic stage (at 21 weeks of age); adrenaline (ng/g wet tissue)
LETO (*n* = 8)	1.2 ± 0.5	1.1 ± 0.3
LETO+RDX (*n* = 8)	1.8 ± 0.4	0.9 ± 0.5
OLETF (*n* = 8)	4.9 ± 0.9**	0.8 ± 0.3
OLETF+RDX (*n* = 8)	5.4 ± 0.6	0.8 ± 0.4

Data are means ± SEM

**p < 0.01 LETO vs OLETF

**Table 2:** Plasma lipid and electrolyte profiles at the diabetic stage (46 weeks of age, protocol 1)

	LETO (*n* = 8)	LETO+RDX (*n* = 8)	OLETF (*n* = 8)	OLETF+RDX (*n* = 8)
Triacylglycerol (mmol/l)	0.56 ± 0.03	0.50 ± 0.07	5.71 ± .059^†††^	3.01 ± 0.56^‡‡‡^
Total cholesterol (mmol/l)	2.71 ± 0.06	1.90 ± 0.07*	5.93 ± 0.18^††^	4.11 ± 0.37^‡‡^
NEFA (mmol/l)	0.35 ± 0.03	0.28 ± 0.04	0.84 ± 0.07^††^	0.47 ± 0.03^‡‡^
Sodium (mmol/l)	141.0 ± 0.8	139.0 ± 0.7	154.0 ± 2.0^†^	145.0 ± 1.2
Potassium (mmol/l)	6.5 ± 0.3	6.4 ± 0.1	6.6 ± 0.3	6.0 ± 0.3

Data are means ± SEM

*p < 0.05 LETO vs LETO+RDX; ^†^ *p* < 0.05, ^††^ *p* < 0.01, ^†††^ *p* < 0.005 LETO vs OLETF; ^‡‡^ *p* < 0.01, ^‡‡‡^ *p* < 0.005 OLETF vs OLETF+RDX

**Table 3:** Plasma lipid and electrolyte profiles at the prediabetic stage (21 weeks of age, protocol 2)

	LETO (*n* = 8)	LETO+RDX (*n* = 8)	OLETF (*n* = 8)	OLETF+RDX (*n* = 8)
Triacylglycerol (mmol/l)	0.40 ± 0.03	0.32 ± 0.03	1.73 ± 0.13^†††^	0.89 ± 0.03**
Total cholesterol (mmol/l)	2.1 ± 0.02	1.9 ± 0.05	2.7 ± 0.08^††^	2.1 ± 0.07**
NEFA (mmol/l)	0.43 ± 0.02	0.41 ± 0.01	0.80 ± 0.02^†††^	0.51 ± 0.01**
Sodium (mmol/l)	133.6 ± 1.9	132.0 ± 2.0	138.5 ± 1.3	134.5 ± 1.0
Potassium (mmol/l)	6.5 ± 0.3	6.0 ± 0.1	6.1 ± 0.2	5.7 ± 0.1

**p < 0.01 OLETF vs OLETF+RDX; ^††^ *p* < 0.01, ^†††^ *p* < 0.005 LETO vs OLETF

As shown in Fig. 1f–i and ESM Fig. 1f–i, SBP, MAP and DBP were significantly higher in OLETF compared with LETO rats, whereas heart rate did not differ among the groups. In protocol 1, RDX significantly decreased BP in OLETF rats, but not in LETO rats. There was a trend towards lower heart rate in OLETF rats given RDX, but these changes were not significant (Fig. 1f–i for SBP, MAP, DPB and heart rate, respectively). In contrast, in protocol 2, RDX did not significantly affect the BP profiles of LETO and OLETF rats at prediabetic stage (ESM Fig. 1f–i for SBP, MAP, DPB and heart rate, respectively). Further details are provided in the ESM Results.

### Glucose metabolism and insulin sensitivity

**Fig. 2:**
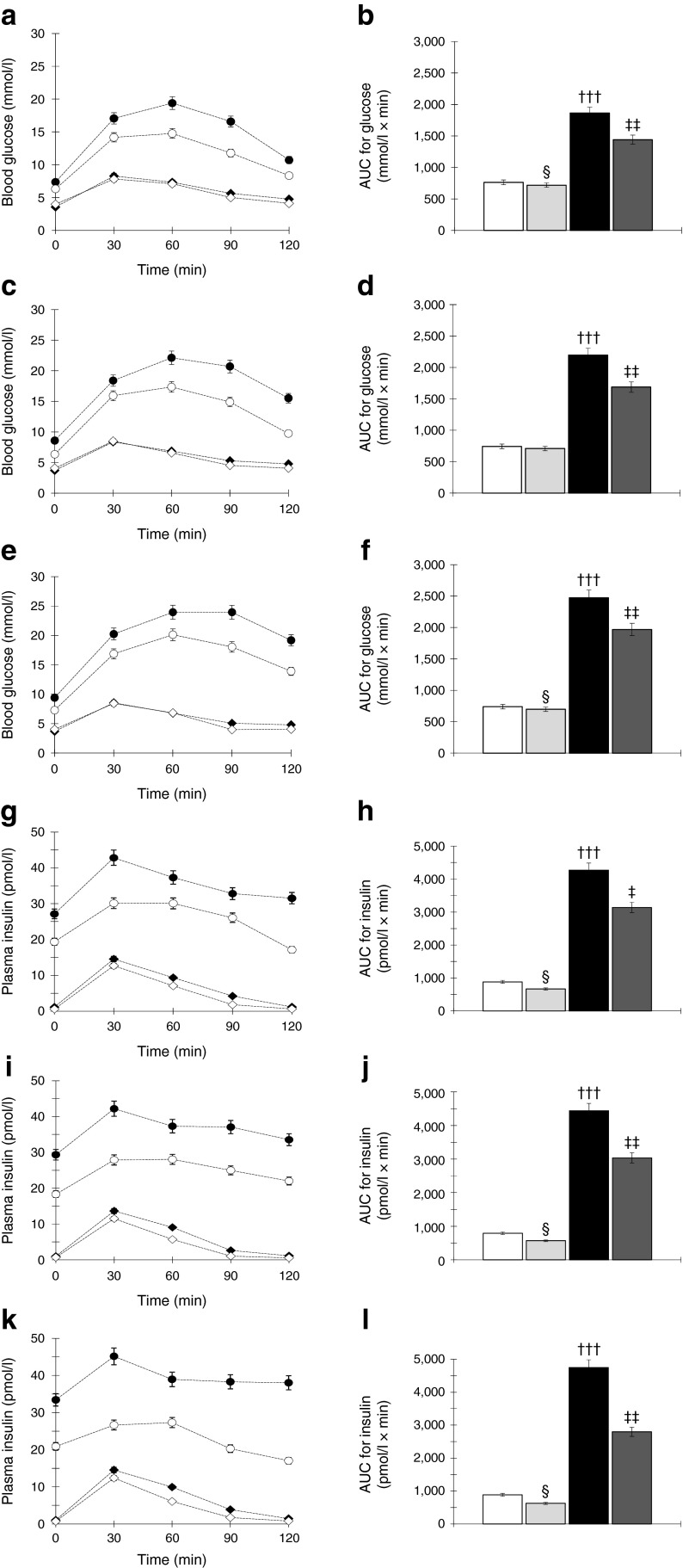
(**a**–**f**) Blood glucose levels and their respective AUCs at 30 (**a**, **b**), 35 (**c**, **d**) and 45 (**e**, **f**) weeks of age during OGTT. OLETF rats show higher glucose levels and AUCs, which are suppressed by RDX. (**g**–**l**) Plasma insulin levels and their AUC at 30 (**g**, **h**), 35 (**i**, **j**) and 45 (**k**, **l**) weeks of age. OLETF rats show higher plasma insulin levels and AUC, which were suppressed by RDX. ^†††^ *p* < 0.005 LETO vs OLETF; ^‡^ *p* < 0.05, ^‡‡^ *p* < 0.01, OLETF vs OLETF+RDX; ^§^ *p* < 0.05, LETO vs LETO+RDX. White bars and white diamonds, LETO group; black bars and black circles, OLETF group; light grey bars and black diamonds, LETO+RDX; dark grey bars and white circles, OLETF+RDX

**Fig. 3:**
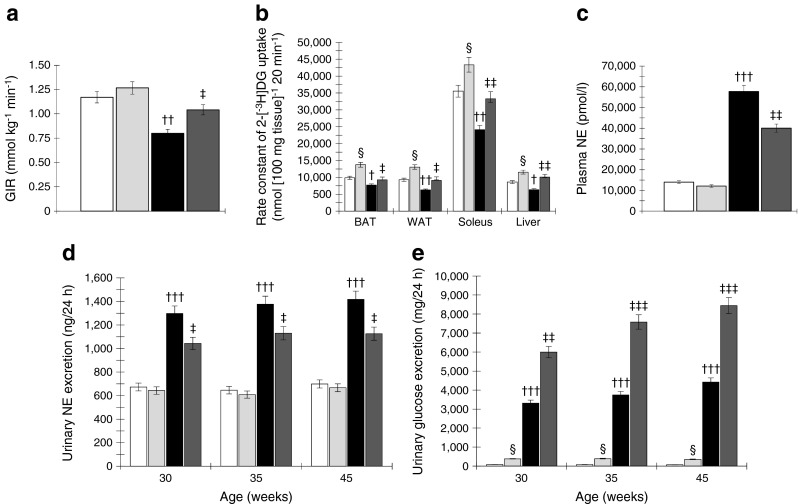
(**a**) OLETF rats show lower GIR during the hyperinsulinaemic–euglycaemic clamp study, which is increased by RDX. (**b**) During rate constant net tissue uptake of 2-[^3^H]DG measurement, OLETF rats show lower glucose uptake in BAT, WAT, soleus muscles and liver tissues compared with LETO rats. RDX improves glucose uptake by these tissues in OLETF rats. (**c**, **d**) OLETF rats show elevated plasma NE levels (**c**) and urinary NE excretion (**d**), which are suppressed by RDX. (**e**) OLETF rats show higher urinary glucose excretion, which is further increased by RDX. ^†^ *p* < 0.05, ^††^ *p* < 0.01, ^†††^ *p* < 0.005 LETO vs OLETF; ^‡^ *p* < 0.05, ^‡‡^ *p* < 0.01, ^‡‡‡^ *p* < 0.005 OLETF vs OLETF+RDX; ^§^ *p* < 0.05 LETO vs LETO+RDX. White bars, LETO group; black bars, OLETF group; light grey bars, LETO+RDX; dark grey bars, OLETF+RDX

In protocol 1, OLETF rats showed significantly elevated glucose and insulin levels after oral administration of glucose compared with LETO rats. RDX attenuated increases in glucose and insulin levels in OLETF rats. The AUC for blood glucose and insulin was significantly greater in OLETF rats, and was significantly suppressed by RDX (Fig. 2a,b and Fig. 2c,d, respectively). Whole body insulin sensitivity was measured using the hyperinsulinaemic–euglycaemic clamp method and OLETF rats showed significantly lowered glucose infusion rate (GIR) compared with LETO rats, which was significantly increased by RDX in OLETF rats (Fig. 3a).

OLETF rats showed significantly lowered in vivo glucose uptake in glucose sensitive tissues such as BAT, WAT, soleus muscle and liver compared with LETO rats. Notably, RDX significantly increased glucose uptake by the peripheral tissues in OLETF rats.

OLETF rats showed elevated plasma NE levels and increased urinary NE excretion during the experimental period compared with age-matched LETO rats (Fig. 3c,d, respectively). RDX significantly attenuated both plasma NE levels and urinary NE excretion. These results indicate that at the diabetic stage, OLETF rats have sympathetic hyperactivity, which was suppressed by RDX. In contrast, RDX markedly increased urinary glucose excretion in both LETO and OLETF rats (Fig. 3e).

In protocol 2, data with a similar glucose metabolism trend to protocol 1 were observed in protocol 2, as shown in ESM Fig. 2a–d and ESM Fig. 3a–e. Further details are provided in the ESM Results.

### Glucose transporters in the kidney

**Fig. 4:**
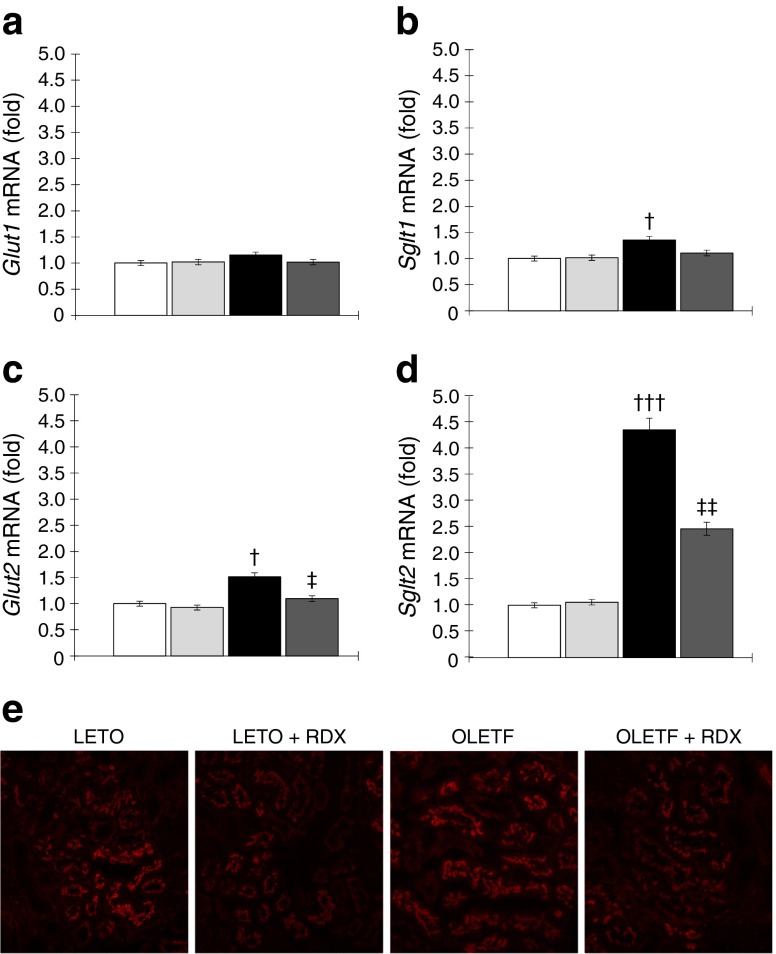
(**a**) *Glut1* mRNA levels are similar among the treatment groups. (**b**, **c**) *Sglt1* (**b**) and *Glut2* (**c**) mRNA expression is upregulated. (**d**) *Sglt2* mRNA level is markedly upregulated in OLETF rats, which is suppressed by RDX. (**e**) Immunofluorescence micrographs of staining with anti-SGLT2 antibody (red fluorescence) (original magnification, ×200). The staining intensity is weaker in OLETF+RDX than OLETF. All mRNA data are expressed as the relative difference in expression compared with LETO rats after normalisation for β-actin expression. ^†^ *p* < 0.05, ^†††^ *p* < 0.005 LETO vs OLETF; ^‡^ *p* < 0.05, ^‡‡^ *p* < 0.01 OLETF vs. OLETF+RDX. White bars, LETO group; black bars, OLETF group; light grey bars, LETO + RDX; dark grey bars, OLETF+RDX

In protocol 1, renal cortical tissue *Glut1* (also known as *Slc2a1*) mRNA levels were similar among the different treatment groups (Fig. 4a). However, *Sglt1* and *Glut2* (also known as *Slc2a2*) mRNA levels were slightly, but significantly, increased in OLETF rats as compared with LETO rats (Fig. 4b,c, respectively). Furthermore, the *Sglt2* mRNA level was markedly upregulated in renal cortical tissue of OLETF compared with LETO rats (Fig. 4d). Increases in *Sglt2* mRNA levels in OLETF rats were significantly decreased by RDX.

Immunohistochemical SGLT2 staining was stronger in OLETF as compared with LETO and OLETF+RDX rats (Fig. 4e). These results showed that in diabetic OLETF rats, renal *Sglt2* expression was upregulated, which was suppressed by RDX. Thus, RDX-induced suppression of renal *Sglt2* expression may lead to reduction in proximal tubular glucose reabsorption, resulting in an increase in urinary glucose excretion.

In protocol 2, similar data on glucose transporter gene and protein expression in the kidney were obtained (ESM Fig. 4a–g). Further details are provided in the ESM Results.

### Glucose transporters expression in the skeletal muscle

The levels of *Glut4* (also known as *Slc2a4*) expression were significantly decreased in the skeletal muscle (soleus muscle) of diabetic (OLETF) rats when compared with control rats (LETO) (ESM Table 3). However, subjecting OLETF rats to RDX restored the expression of *Glut4* in the skeletal muscle when compared with untreated diabetic (OLETF) rats. Subjecting LETO rats to RDX produced no significant changes in the expression of *Glut4* in skeletal muscle when compared with normal control (LETO) rats. Similar results were observed in prediabetic stage rats as shown in ESM Table 3.

### Renal functional and histological changes

**Fig. 5:**
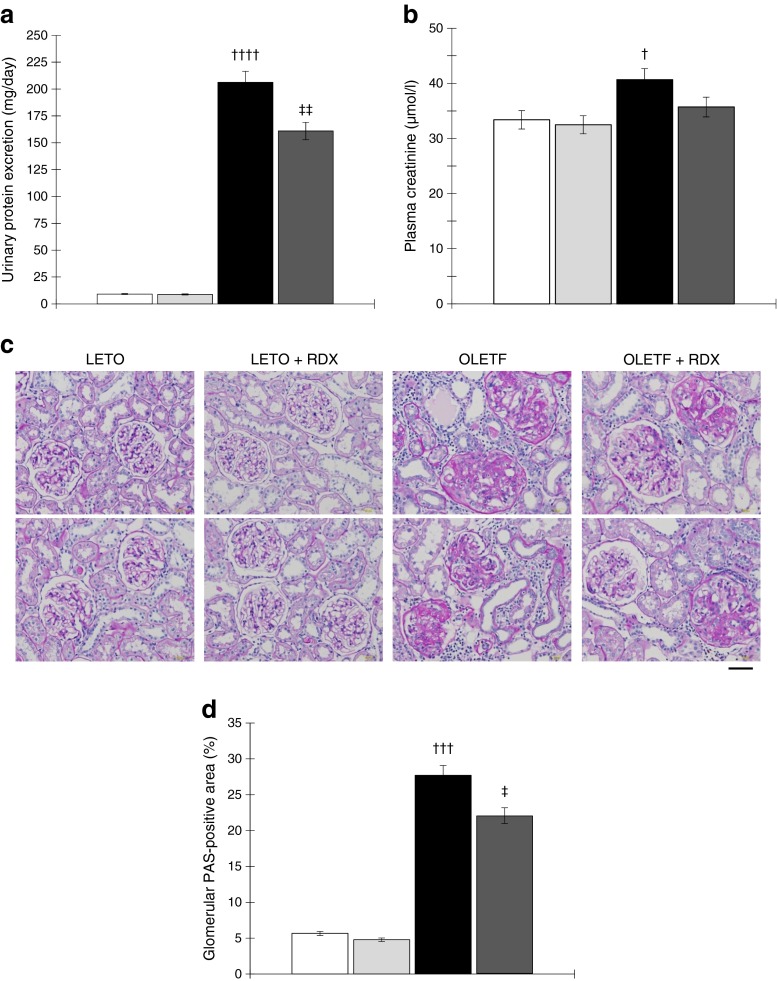
(**a**) OLETF rats show overt proteinuria, which is reduced by RDX. (**b**) Plasma creatinine concentration was slightly elevated in OLETF rats and is attenuated by RDX. (**c**) Representative micrographs of PAS-stained renal sections. (**d**) PAS-positive area within the total glomerular area. RDX partially reduced PAS-positive area. ^†^ *p* < 0.05, ^†††^ *p* < 0.005, ^††††^ *p* < 0.001 LETO vs OLETF; ^‡^ *p* < 0.05, ^‡‡^ *p* < 0.01 OLETF vs OLETF+RDX. Scale bar, 50 μm. White bars, LETO group; black bars, OLETF group; light grey bars, LETO+RDX; dark grey bars, OLETF+RDX

In protocol 1, OLETF rats developed proteinuria age-dependently. RDX significantly reduced proteinuria in OLEFT rats (Fig. 5a). Plasma creatinine concentration was significantly higher in OLETF rats compared with LETO rats, which was lowered by RDX (Fig. 5b). Renal cortical sections showed increased glomerular PAS-positive area in OLETF rats compared with LETO rats. RDX significantly decreased glomerular PAS-positive area (Fig. 5c,d).

In protocol 2, OLETF rats showed proteinuria and increased PAS-positive area compared with LETO rats. However, RDX did not significantly affect these variables (ESM Fig. 5a–d) at the prediabetic stage. Further details are provided in the ESM Results.

### Cell culture experiments

**Fig. 6:**
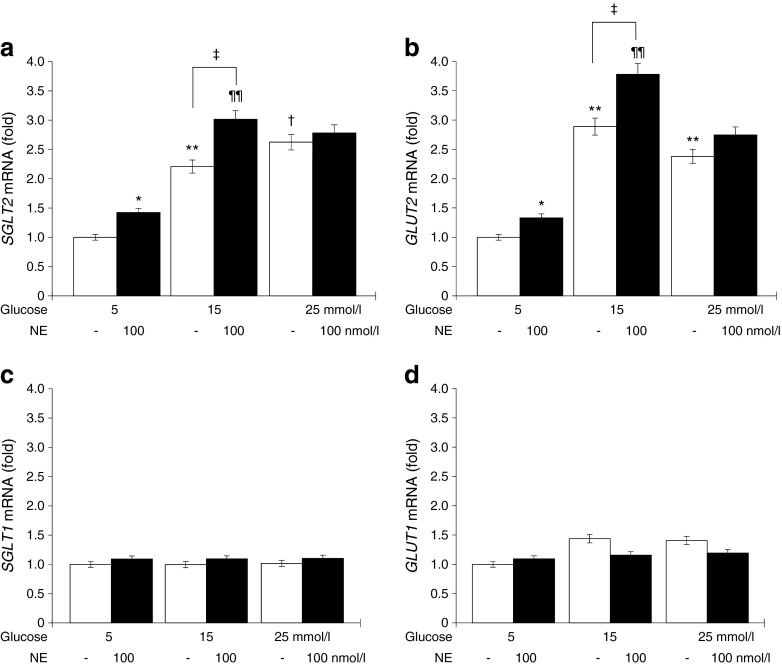
(**a**) Treatment with high glucose for 12 h upregulated *SGLT2* mRNA expression, which was further enhanced by exposure to NE in HK2 human kidney proximal tubule epithelial cells. (**b**) *GLUT2* mRNA expression was also upregulated by high glucose treatment, and was further enhanced by exposure to NE. (**c**, **d**) In contrast, *SGLT1* (**c**) and *GLUT1* (**d**) mRNA expression remained unaltered after high glucose and/or high glucose plus NE treatment. Data are expressed as the relative difference in expression compared with 5 mmol/l glucose after normalisation for β-actin expression. *p < 0.05, **p < 0.01 vs 5 mmol/l glucose; ^¶¶^ *p* < 0.01 vs 5 mmol/l glucose + NE; ^†^ *p* < 0.05 vs 15 mmol/l glucose. ^‡^ *p* < 0.05, as indicated

To further confirm the possible contribution of sympathetic nervous system to renal *SGLT2* (also known as *SLC5A2*) expression, we performed in vitro studies using immortalised human kidney proximal tubular epithelial HK2 cells. Treatment with high glucose for 12 h upregulated *SGLT2* mRNA expression, which was further enhanced by exposure to NE (Fig. 6a). *GLUT2* (also known as *SLC2A2*) mRNA expression was also upregulated by high glucose treatment, and was further enhanced by exposure to NE (Fig. 6b). In contrast, *SGLT1* (also known as *SLC5A1*) and *GLUT1* (also known as *SLC2A1*) mRNA expressions remained unaltered after high glucose and/or high glucose plus NE treatment (Fig. 6c,d). Similarly, exposure to high glucose plus NE for 24 h significantly upregulated *SGLT2* gene expression in HK2 cells (ESM Fig. 6a–d).

## Discussion

In the present study, we demonstrate that during the development of type 2 diabetes, RDX attenuates systemic and regional sympathetic hyperactivity, and substantially reduces glucose intolerance and insulin insensitivity through two different mechanisms, specifically: (1) improvement of glucose uptake by peripheral tissues; and (2) enhancement of urinary glucose excretion by suppression of renal *Sglt2* overexpression.

A growing body of evidence indicates there is inappropriate activation of the sympathetic nervous system during the development of insulin resistance [38], the metabolic syndrome [39] and diabetes [5, 8]. Sympathetic hyperactivity could be responsible for lower glucose uptake by peripheral tissues, due to a reduction in blood flow with increased peripheral NE release [40]. A direct relationship between muscle sympathetic hyperactivity and insulin resistance has also been indicated [41]. In the present study, obese type 2 diabetic and prediabetic OLETF rats showed a reduction in tissue glucose uptake with systemic sympathetic hyperactivity as confirmed by increases in plasma and urine NE levels. Furthermore, RDX decreased systemic NE levels and improved tissue glucose uptake in these animals. Thus, the present data are consistent with concepts based on previous clinical studies [17, 18], namely that the systemic sympathetic nervous system plays an important role in glucose metabolism by regulating tissue glucose uptake during the development of type 2 diabetes.

**Fig. 7:**
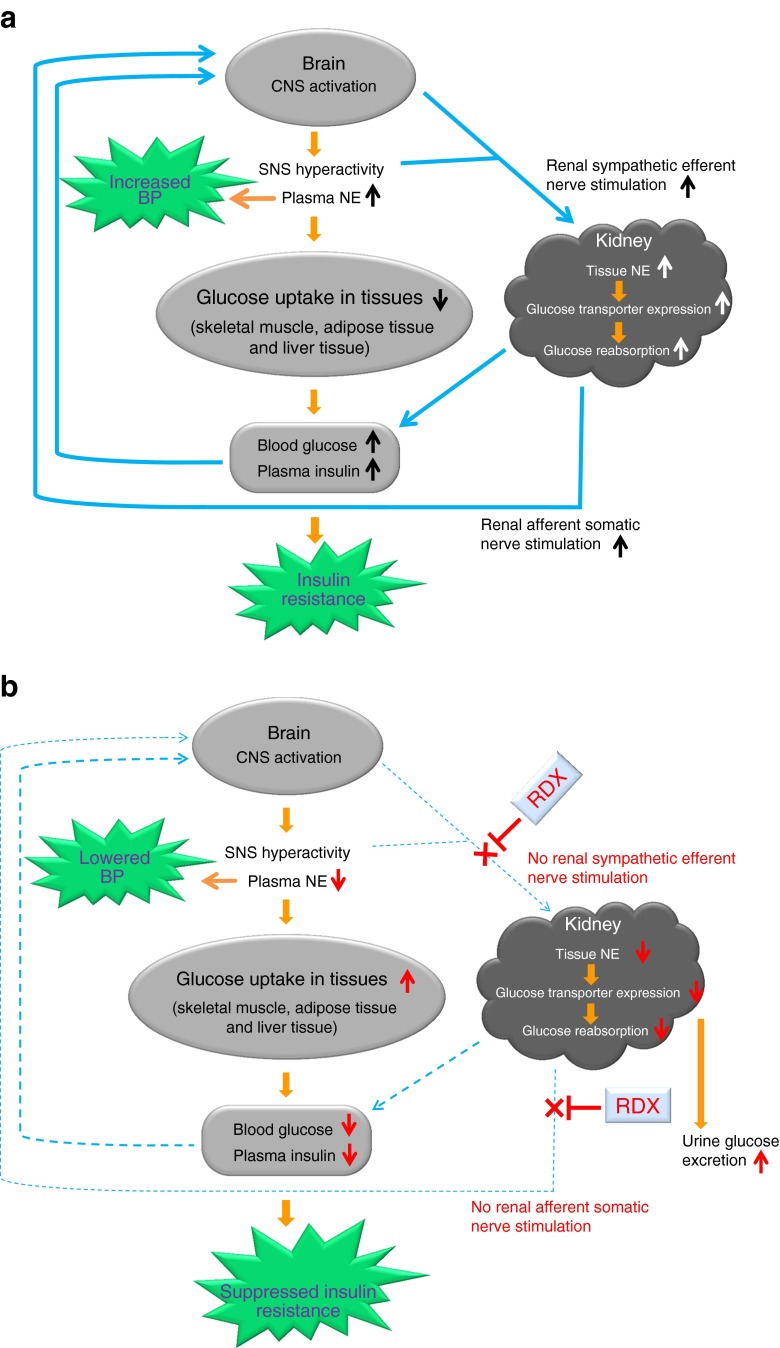
(**a**) Schematic diagram summarising the proposed mechanisms by which renal sympathetic nerves contribute to glucose metabolism during the development of type 2 diabetes. (**b**) Effects of RDX on renal glucose metabolism. CNS, central nervous system, SNS; sympathetic nervous system

It has also been suggested that insulin acts as a potential mediator of sympathetic overdrive in hypertension and other cardiovascular complications [1, 3]. Alternatively, intrarenal sympathetic hyperactivity may cause increased renal afferent nerve signalling leading to further stimulation of the central sympathetic nervous system, thus initiating a vicious cycle (Fig. 7a). RDX breaks this vicious cycle resulting in suppression of the sympathetic nervous system, and thereby improved glucose metabolism and insulin sensitivity in obese type 2 diabetic rats (Fig. 7b). These data suggest an interaction among insulin resistance, obesity and the central nervous system. The most plausible interpretation of the above results is in clinical use of a centrally acting sympatholytics drug, which is known to reduce central sympathetic activity [42], reduce efferent sympathetic nerve traffic to the kidney, and suppress glucose intolerance and increase skeletal muscle blood flow with less glycogenolysis [1, 43]. Although centrally acting sympatholytics have some beneficial effects in clinics [44, 45], these drugs are nonspecific and limited by their adverse effects on insulin resistance and an unawareness of hypoglycaemia in patients with diabetes [45]. Adverse effects of blockade of β2-receptors on glucose metabolism have also been recognised [46, 47]. In contrast to nonselective β-blockers, β1-selective blockers appear to be without relevant influence on glucose metabolism [47, 48]. However, in some studies, adverse effects of β1-selective β-blockers have been described [49]. Glycogenolysis and gluconeogenesis in liver are stimulated through β2-receptors [50]. Blockade of these receptors could prolong recovery-time from hypoglycaemia. Under nonselective β-blocker treatment, such prolongation of hypoglycaemia has been described [51]. Therefore, suppressing the sympathetic nervous system directly by RDX seems to be a better option for diabetes. Furthermore, hypertension, glucose intolerance and insulin insensitivity regularly coincide, with sympathetic hyperactivity being an obvious but not the only link among these, but also other pathological conditions [2].

The potential role of the kidney in the regulation of blood glucose levels has been well documented in earlier studies [52]. SGLT2 is a high-capacity, low-affinity glucose transporter located in the early convoluted segment of the renal proximal tubule, where luminal glucose is abundant [53–55]. SGLT2 reabsorbs approximately 90% of filtered renal glucose [56, 57] by coupling glucose transport to the electrochemical sodium gradient [53–55]. SGLT2 inhibitors reduce renal glucose reabsorption and promote urinary glucose excretion, thus lowering glucose blood levels. In the present study, renal *Sglt2* gene and SGLT2 protein levels were significantly increased in obese type 2 diabetic OLETF rats. Interestingly, the present study also demonstrated for the first time that RDX attenuates *Sglt2* overexpression in the kidney. These findings are consistent with the concept that during the development of type 2 diabetes, intrarenal sympathetic hyperactivity enhances renal *Sglt2* expression, resulting in increased glucose reabsorption. This concept was further evaluated by an in vitro cell culture study. In cultured human proximal tubular HK2 cells, high glucose conditions significantly increased *SGLT2* gene expression, which was further augmented by treatment with NE. Collectively, the present study demonstrates that RDX improves glucose metabolism by inhibiting inappropriately augmented renal *Sglt2* expression, and results in a subsequent glycosuria during the development of type 2 diabetes. These data suggest that RDX is a logical approach not only for the treatment of hypertension and insulin resistance, but also for other clinical adverse consequences manifested by sympathetic hyperactivity. Furthermore, Dominik Linz et al [5] previously reported the effects of RDX in SHRs-ob rats when hypertension, the metabolic syndrome and nephropathy are already established, and showed that even at this later stage of renal and cardiac remodelling, RDX attenuated the further progression of hypertension, renal and cardiac damage, suggesting the pivotal role of renal sympathetic activation in these pathophysiological conditions and of RDX for the management of diabetes in obese spontaneously hypertensive rats. However, there are some limitations of current clinical studies for RDX. First, the SYMPLICITY HTN-3 clinical trial did not show significant reduction in BP in resistant hypertensive patients in a randomised, controlled trial [16]. Moreover, the finding that RDX improves glucose metabolism in resistant hypertensive patients [17, 18] has not been confirmed in controlled trials and has not been separated from its BP-lowering effects.

In the present study, there are several limitations as it is still unclear that the beneficial effects of RDX are due to ablation of renal efferent vs afferent nerves. It is important to note that glucose tolerance, insulin sensitivity and urinary protein excretion are still high in rats subjected to RDX compared with control rats. This may be due to other known factors [2] that are involved in insulin resistance in this model. Alternatively, it is possible that the degree of diabetes is too severe to fully reverse changes by RDX. Further studies are needed to address such issues.

We previously showed that RDX did not change BP in rats subjected to aortic regurgitation, but elicited beneficial effects on cardio-renal syndrome [15]. Other studies have also suggested that RDX ameliorates the incidence of stroke and brain injury in hypertensive rats, independent of BP changes [58]. In the present study, RDX decreased BP and improved glucose metabolism in type 2 diabetic OLETF rats. However, in prediabetic animals, RDX also significantly improved insulin resistance without changing BP. These data suggest that the beneficial effects of RDX on glucose intolerance and insulin resistance cannot be explained solely by its BP-lowering effects.

## Conclusions

Here, we propose the novel concept of a connection between renal glucose metabolism and the sympathetic nervous system (Fig. 7a). The activities of the systemic nervous system and renal *SGLT2* are regulated by afferent and efferent renal nerve activities, respectively, both of which contribute to glucose intolerance and insulin resistance during the development of type 2 diabetes. RDX results in a suppression of the sympathetic nervous system and subsequent improvement of glucose metabolism (Fig. 7b). Thus, the present data may provide a new insight not only into the treatment of hypertensive diabetic patients, but also the pathophysiology of insulin resistance manifested by sympathetic hyperactivity.

## Electronic supplementary material

ESM Methods (PDF 112 kb)

ESM Results (PDF 39 kb)

ESM Fig. 1 (PDF 117 kb)

ESM Fig. 2 (PDF 139 kb)

ESM Fig. 3 (PDF 24 kb)

ESM Fig. 4 (PDF 208 kb)

ESM Fig. 5 (PDF 381 kb)

ESM Fig. 6 (PDF 36 kb)

ESM Table 1 (PDF 24 kb)

ESM Table 2 (PDF 24 kb)

ESM Table 3 (PDF 8 kb)

## Abbreviations

2-[^3^H]DG: 2-[^3^H]Deoxy-d-glucose
BAT: Brown adipose tissue
DBP: Diastolic BP
GIR: Glucose infusion rate
LETO: Long-Evans Tokushima Otsuka rat
MAP: Mean arterial pressure
NE: Norepinephrine (noradrenaline)
OLETF: Otsuka Long-Evans Tokushima fatty rat
PAS: Periodic acid-Schiff
RDX: Renal sympathetic denervation
SBP: Systolic BP
SGLT2: Sodium-glucose cotransporter 2
WAT: Retroperitoneal white adipose tissues
