# Supplementary material for: Role of the renal sympathetic nerve in renal glucose metabolism during the development of type 2 diabetes in rats
Source: Diabetologia. 2015 Oct 8;58(12):2885–98. doi: 10.1007/s00125-015-3771-9 (PMC4630257; doi:10.1007/s00125-015-3771-9)
Supplement: Supplementary file 1 — (PDF 112 kb) [file 125_2015_3771_MOESM1_ESM.pdf]

Supplement Methods

Blood pressure measurement

Systolic blood pressure (SBP) was monitored in conscious rats by radio-telemetry system (Data Sciences International, St. Paul, MN, USA) in conscious rats as described previously.[1, 2]

Oral Glucose Tolerance Test (OGTT)

OGTT was performed as described previously.[3, 4] In brief, animals were food restricted overnight and OGTT was carried out with a 2 g/kg body weight glucose feeding by gavage. Blood samples were collected from a cut at the tip of the tail before and 30, 60, 90 and 120 min after the glucose feeding, and blood glucose and plasma insulin levels were measured.

Hyperinsulinemic-Euglycemic Clamp Study

Rats were anesthetized with isoflurane. Then, both femoral veins were exposed and a catheter was inserted for infusion of glucose and insulin, respectively. Another catheter was inserted into the femoral artery for blood sampling. After a 30 min stabilization period, a 120 min hyperinsulinemic-euglycemic clamp was performed according to previous reports.[5, 6] Hyperinsulinemic-euglycemic clamp study was performed at 20 weeks of age for protocol-2 and at 45 weeks of age for protocol-1 using separate sets of animals (*n* = 6 for each treatment group).

Telemetric Sensor Implantation

Blood pressure profiles (mean arterial pressure; MAP, SBP and diastolic blood pressure; DBP) and heart rate (HR) were assessed by a radio-telemetry system in conscious rats as described previously[1, 2] in a separate set of animals (*n* = 5 for each treatment group). Briefly, under

isoflurane anesthesia, a telemetry BP probe (model TA11PAC40, Data Sciences International, St. Paul, MN, USA) was positioned intra-abdominally and secured to the ventral abdominal muscle with a sensor catheter inserted into the right femoral artery, and the tip of the catheter was in the abdominal aorta caudal to the renal arteries. The telemetry signals were processed and digitized as radio frequency data, which were recorded and stored using the Dataquest IV system (Data Sciences International). After one week recovery, rats were placed above the telemetric receivers, and blood pressure and HR were recorded at 5 min intervals continuously for twenty-four hours at 13, 17, 20 and 24 weeks of age in protocol-1 and 39, 41, 43 and 45 weeks of age in protocol-2.

Measurement of Rate Constant of Net Tissue Uptake of 2-[^{3}H]Deoxy-D-Glucose

In vivo uptake of 2-[^{3}H]deoxy-D-glucose (2-[^{3}H]DG) in peripheral tissues was measured as described previously[7, 8] in separate groups of animals (*n* = 6 for each treatment group). Brown adipose tissue (BAT), retroperitoneal white adipose tissues (WAT), skeletal muscles (soleus muscles) and liver were rapidly dissected and weighed. The rate constant of net tissue uptake of 2-[^{3}H]DG was calculated as described previously.[9] Briefly, to establish the rate constant of basal glucose uptake, rats were fasted overnight and 50 µCi of 2-deoxy-D-[^{3}H]glucose (2-[^{3}H]DG; Perkin Elmer Life and Analytical Sciences, Boston, USA) and 5 µCi of [^{14}C]sucrose (Perkin Elmer Life and Analytical Sciences) per rat dissolved in 0.4 ml of saline were injected via the caudal vein. Blood was taken 0, 10, 15 and 20 min after injection of radioactive tracers. The animals were killed by cervical dislocation 20 min after injection, and skeletal muscles (soleus), BAT, WAT and liver were rapidly dissected and weighed. The rate constant of the 2-[^{3}H]DG uptake was calculated. For measurement of insulin-stimulated glucose uptake, the fed

rats were injected with 0.5 U/kg of human insulin intraperitoneally. Ten min after insulin injection, the injection of the radioactive tracers, dissection of the tissues, and calculation of the rate constant of 2-[^{3}H]DG uptake were performed as per the basal glucose uptake measurement study. The *in vivo* glucose uptake study was performed at 45 weeks of age for protocol-1 and at 20 weeks of age for protocol-2.

Sample Collection

At the end of each observation period, blood was collected and kidneys perfused with an isotonic saline under anesthesia with sodium pentobarbital (65 mg/kg, i.p.). Then, kidney tissues were harvested and fixed in 10% buffered paraformaldehyde or embedded in Tissue-Tek OCT compound (Sakura Finetek Japan Co., Ltd., Tokyo, Japan), and remaining tissues were snap-frozen in liquid nitrogen for western blot analysis and NE measurement. Small amounts of renal cortical tissues were collected in RNAlater (Sigma-Aldrich, Inc, St. Louis, MO, USA) and stored overnight at 4°C. RNAlater-treated samples were subsequently snap-frozen in liquid nitrogen for RNA extraction and reverse transcription-polymerase chain reaction (RT-PCR) analysis.

Tissue Fixation and Immunofluorescence Study

Tissue fixation and immunofluorescence detection of *SGLT2* were performed as described previously.[10] Briefly, before staining with the antibody, 4 μm frozen sections were rehydrated in PBS for 10 min, heated in 10 mmol/l citrate buffer, pH 6, for 20 min (5 min x 4 cycles at 800 W) in a microwave oven, followed by cooling to room temperature in the same buffer for approximately 1 h. Subsequently, sections were incubated in a wet chamber with 0.5% Triton X-100 (in PBS) for 5 min, rinsed with PBS (5 min x 5 times), incubated for 20 min with 1% BSA

in PBS, incubated with the *SGLT2* antibody (diluted 1:500 with PBS) overnight in a refrigerator, rinsed with PBS (5 min x 4 times). Sections were rinsed with PBS (5 min x 4 times), and mounted in a fluorescence fading retardant with DAPI (Vectashield, Vector Laboratories, Burlingame, CA, USA), and prepared for microscopy. The stained sections were examined and images were obtained by confocal laser-scanning fluorescence microscopy (Radiance2100; Bio-Rad Laboratories, Hercules, CA, USA).

Other Analytical Procedures

Plasma, urine and renal cortical tissue NE levels were measured as described previously.[11, 12] Plasma levels of non-esterified fatty acid (NEFA), tryacylglycerol (TG), total cholesterol (TCho), creatinine, electrolytes and blood HbA1c were measured using an automatic analyzer (Model 7020, HITACHI, Tokyo, Japan). Blood glucose was measured with a glucometer (Sanwa-Kagaku Co., Ltd., Nagoya, Japan). Plasma insulin (Rat Insulin ELISA kit; Shibayagi, Shibukawa, Japan), glucose (Glucose Assay Kit, BioVision, CA, USA) and urine protein (micro TP-test; Wako Pure Chemical Industries, Ltd., Osaka, Japan) concentrations were measured using commercially available assay kits.

Cell Culture Experiments

HK2 cells (immortalized human kidney proximal tubule epithelial cells) were cultured in DMEM supplemented with 10% fetal bovine serum (FBS) (PAA Laboratories, GE Healthcare Bio-Sciences, Uppsala, Sweden; A15-701), penicillin (50 U/ml) and streptomycin (50 µg/ml; Life Technologies, Carlsbad, CA, USA; 15070)[12]. Cells were grown in a humidified incubator at 37°C in an atmosphere of 5% CO_2 and 95% air. After reaching 40% confluence in a 6-well plate,

cells were serum deprived for 24 h before experimental manipulation. All experiments were performed under serum-free conditions and the cells remained viable in this condition in a nonproliferating state. Quiescent cells were then treated with normal glucose medium (5 mmol/l; DMEM, Sigma-Aldrich, St Louis, MO, USA; D5546) as well as high glucose (15 mmol/l; Mixture of DMEM, Sigma-Aldrich, D5546 and DMEM, Sigma-Aldrich, D5796 as 1:1, and 25 mmol/l; DMEM, Sigma-Aldrich, D5796) medium with or without 100 nmol/l NE (Noradrenalin, 0.1%, Daiichi-Sankyo, Tokyo, Japan) for 12 and 24 h.

Following 12 or 24 h treatment with normal and/or high glucose medium with or without NE, cells were washed with cold PBS twice and then 500 μl Isogen (Molecular Research Center, Inc, Cincinnati, OH, USA; 311-02501) was added in each well of a 6-well plate and the cells collected. mRNA was extracted from cells using the phenol-chloroform extraction method. The expression levels of *β*-actin, sodium-glucose cotransporter 1 (*SGLT1*), *SGLT2*, glucose transporter 1 (*GLUT1*) and *GLUT2* were analyzed by RT-PCR using an ABI Prism 7,000 with Power SYBR Green PCR Master Mix (Applied Biosystems, Foster City, CA, USA) as previously described.[12, 13] The oligonucleotide primer sequences for human *β*-actin, *GLUT1*, *GLUT2*, *SGLT1*, and *SGLT2* are listed in Supplemental Table 2. Data are expressed as the relative difference in expression compared with 5 mmol/l glucose after normalization for *β*-actin expression.

References

1. Prysyazhna O, Rudyk O, Eaton P (2012) Single atom substitution in mouse protein kinase G eliminates oxidant sensing to cause hypertension. Nat Med 18: 286-290
2. Zhang YC, Bui JD, Shen L, Phillips MI (2000) Antisense inhibition of beta(1)-adrenergic receptor mRNA in a single dose produces a profound and prolonged reduction in high blood pressure in spontaneously hypertensive rats. Circulation 101: 682-688
3. Henriksen EJ, Jacob S, Kinnick TR, Teachey MK, Krekler M (2001) Selective angiotensin II receptor antagonism reduces insulin resistance in obese Zucker rats. Hypertension 38: 884-890
4. Rafiq K, Hitomi H, Nakano D, Ichihara A, Nishiyama A (2011) Possible involvement of the (pro)renin receptor-dependent system in the development of insulin resistance. Front Biosci (Schol Ed) 3: 1478-1485
5. Kraegen EW, James DE, Bennett SP, Chisholm DJ (1983) In vivo insulin sensitivity in the rat determined by euglycemic clamp. Am J Physiol 245: E1-7
6. Ogihara T, Asano T, Ando K, et al. (2002) Angiotensin II-induced insulin resistance is associated with enhanced insulin signaling. Hypertension 40: 872-879
7. Shiuchi T, Iwai M, Li HS, et al. (2004) Angiotensin II type-1 receptor blocker valsartan enhances insulin sensitivity in skeletal muscles of diabetic mice. Hypertension 43: 1003-1010
8. Shiuchi T, Nakagami H, Iwai M, et al. (2001) Involvement of bradykinin and nitric oxide in leptin-mediated glucose uptake in skeletal muscle. Endocrinology 142: 608-612
9. Sudo M, Minokoshi Y, Shimazu T (1991) Ventromedial hypothalamic stimulation enhances peripheral glucose uptake in anesthetized rats. Am J Physiol 261: E298-303

10. Sabolic I, Vrhovac I, Eror DB, et al. (2012) Expression of Na+-D-glucose cotransporter SGLT2 in rodents is kidney-specific and exhibits sex and species differences. Am J Physiol Cell Physiol 302: C1174-1188

11. Nakashima A, Matsuoka H, Yasukawa H, et al. (1996) Renal denervation prevents intraglomerular platelet aggregation and glomerular injury induced by chronic inhibition of nitric oxide synthesis. Nephron 73: 34-40

12. Rafiq K, Noma T, Fujisawa Y, et al. (2012) Renal sympathetic denervation suppresses de novo podocyte injury and albuminuria in rats with aortic regurgitation. Circulation 125: 1402-1413

13. Rafiq K, Nakano D, Ihara G, et al. (2011) Effects of mineralocorticoid receptor blockade on glucocorticoid-induced renal injury in adrenalectomized rats. J Hypertens 29: 290-298
