# Supplementary material for: Role of the renal sympathetic nerve in renal glucose metabolism during the development of type 2 diabetes in rats
Source: Diabetologia. 2015 Oct 8;58(12):2885–98. doi: 10.1007/s00125-015-3771-9 (PMC4630257; doi:10.1007/s00125-015-3771-9)
Supplement: Supplementary file 2 — (PDF 39 kb) [file 125_2015_3771_MOESM2_ESM.pdf]

Supplement Results from Protocol-2 (at the Prediabetic Stage)

General* variables *and Blood Pressure Profile

LETO and OLETF rats were subjected to RDX at 6 weeks of age. OLETF rats have significantly higher kidney tissue NE levels compared with age matched LETO rats (ESM Fig.1a), however RDX resulted in decreases in kidney NE levels to below the detectable range. During the experimental period, OLETF rats showed significantly higher body weight gain and daily food intake compared with LETO rats. However, RDX does not affect these variables in LETO or in OLETF rats (ESM Fig. 1b-c) at the prediabetic stage. Fasting blood glucose and HbA_{1c} levels at 20 weeks of age (14 weeks after RDX) were significantly higher in OLETF rats compared with LETO rats (ESM Fig. 1d-e), which were lowered by RDX in OLETF rats. At 20 weeks of age, plasma insulin levels were significantly higher in OLETF rats compared with LETO rats (1.07 ± 0.74 and 5.03 ± 1.70 pmol/l, $p < 0.02$, respectively), which was attenuated by RDX (3.41 ± 1.50 pmol/l, $p < 0.15$). Plasma tryacylglycerol, total cholesterol and NEFA were significantly higher in OLETF rats compared with LETO rats, and were lowered by RDX in OLETF rats (Table 3). Plasma sodium and potassium levels were similar between the treatment groups (Table 3).

RDX to diabetic (OLETF) rats showed non-significant tread to increase sodium excretion (OLETF is 0.27±0.03 mmol/day, OLETF+RDX is 0.88±0.2 mmol/day). Similar results were observed in non-diabetic (LETO) rats (LETO is 0.32±0.03 mmol/day, LETO +RDX is 0.57±0.1 mmol/day).

As shown in ESM Fig. 1f-i, 24 h SBP, MAP DBP and heart rate measured by telemetry system were significantly elevated in OLETF compared with LETO rats. However, RDX did not significantly affect the blood pressure profiles and HR at the prediabetic stage (ESM Fig. 1f-i for SBP, MAP, DPB and heart rate, respectively).

Glucose Metabolism and Insulin Sensitivity

We performed OGTT at 14 and 20 weeks of age (8 and 14 weeks after RDX, respectively), in which both blood glucose and insulin concentrations were determined and their respective areas under the curve (AUC) calculated. Glucose and insulin responses during OGTT in all groups are shown in ESM Fig. 2a-d. OLETF rats showed significantly higher glucose and insulin levels after oral administration of glucose compared with LETO rats. RDX attenuated increased glucose and insulin levels observed in OLETF rats. AUC for blood glucose and insulin were also substantially greater in OLETF rats, both of which were significantly suppressed by RDX (ESM Fig. 2a-b for glucose and its AUC at 14 and 20 weeks of age, respectively. ESM Fig. 2c-d for insulin and its AUC at 14 and 20 weeks of age, respectively).

We also examined whole body insulin sensitivity using the hyperinsulinemic-euglycemic clamp test (ESM Fig. 3a). OLETF rats showed significantly lowered glucose infusion rate (GIR) compared with LETO rats at 20 weeks of age, which was significantly increased by RDX in OLETF rats.

We investigated *in vivo* glucose uptake in brown adipose tissue (BAT), white adipose tissue (WAT), soleus muscles and liver tissue. OLETF rats at 20 weeks of age showed significantly lowered glucose uptake by BAT, WAT, soleus muscle and liver compared with LETO rats. RDX significantly increased glucose uptake by these peripheral tissues in OLETF rats (ESM Fig. 3b).

OLETF rats at 21 weeks of age showed elevated plasma NE levels, and increased urinary NE excretion compared with LETO rats (ESM Fig. 3c-d, respectively), suggesting that OLETF rats show sympathetic hyperactivity at the prediabetic stage. RDX significantly decreased plasma

and urine NE levels in OLETF rats.

Interestingly, OLETF + RDX rats showed overt glycosuria compared with non denervated OLETF rats (ESM Fig. 3e). These results indicate that the renal sympathetic nerve plays an important role in regulating glucose handling in the kidney.

Glucose Transporters Expression in the Kidney

Renal cortical tissue *Glut1* (also known as *Slc2a1*) and *Sglt1* mRNA levels were similar between the treatment groups (ESM Fig. 4a-b). However, *Glut2* (also known as *Slc2a2*)and *Sglt2* mRNA levels were significantly increased in OLETF rats compared with LETO rats (ESM Fig. 4c-d). Increases in *Glut2* and *Sglt2* mRNA levels were suppressed by RDX.

Immunofluorescence analysis with a SGLT2 specific antibody was performed. The staining for SGLT2 was weaker in OLETF + RDX rats (ESM Fig. 4e) compared with OLETF rats. These results suggest that OLETF rats showed upregulation of *Sglt2* expression at the prediabetic stage, which was suppressed by RDX. Thus the effects of RDX may induce suppression of glucose reabsorption by the renal proximal tubules, resulting in marked glycosuria in OLETF rats.

Renal Functional and Structural Parameters

OLETF rats at 20 weeks of age showed increased urinary protein excretion compared with LETO rats, which was reduced by RDX (ESM Fig. 5a). However, plasma creatinine concentration remained similar between the treatment groups (ESM Fig. 5b). Structural changes in kidney tissue were evaluated by PAS staining. Data showed increased PAS positive area and glomerular hypertrophy in OLETF rats compared with LETO rats. However, RDX did not significantly affect these parameters at the prediabetic stage (ESM Fig. 5c-d).
