# Supplementary material for: Role of the renal sympathetic nerve in renal glucose metabolism during the development of type 2 diabetes in rats
Source: Diabetologia. 2015 Oct 8;58(12):2885–98. doi: 10.1007/s00125-015-3771-9 (PMC4630257; doi:10.1007/s00125-015-3771-9)
Supplement: Supplementary file 3 — (PDF 117 kb) [file 125_2015_3771_MOESM3_ESM.pdf]

ESM Fig. 1.

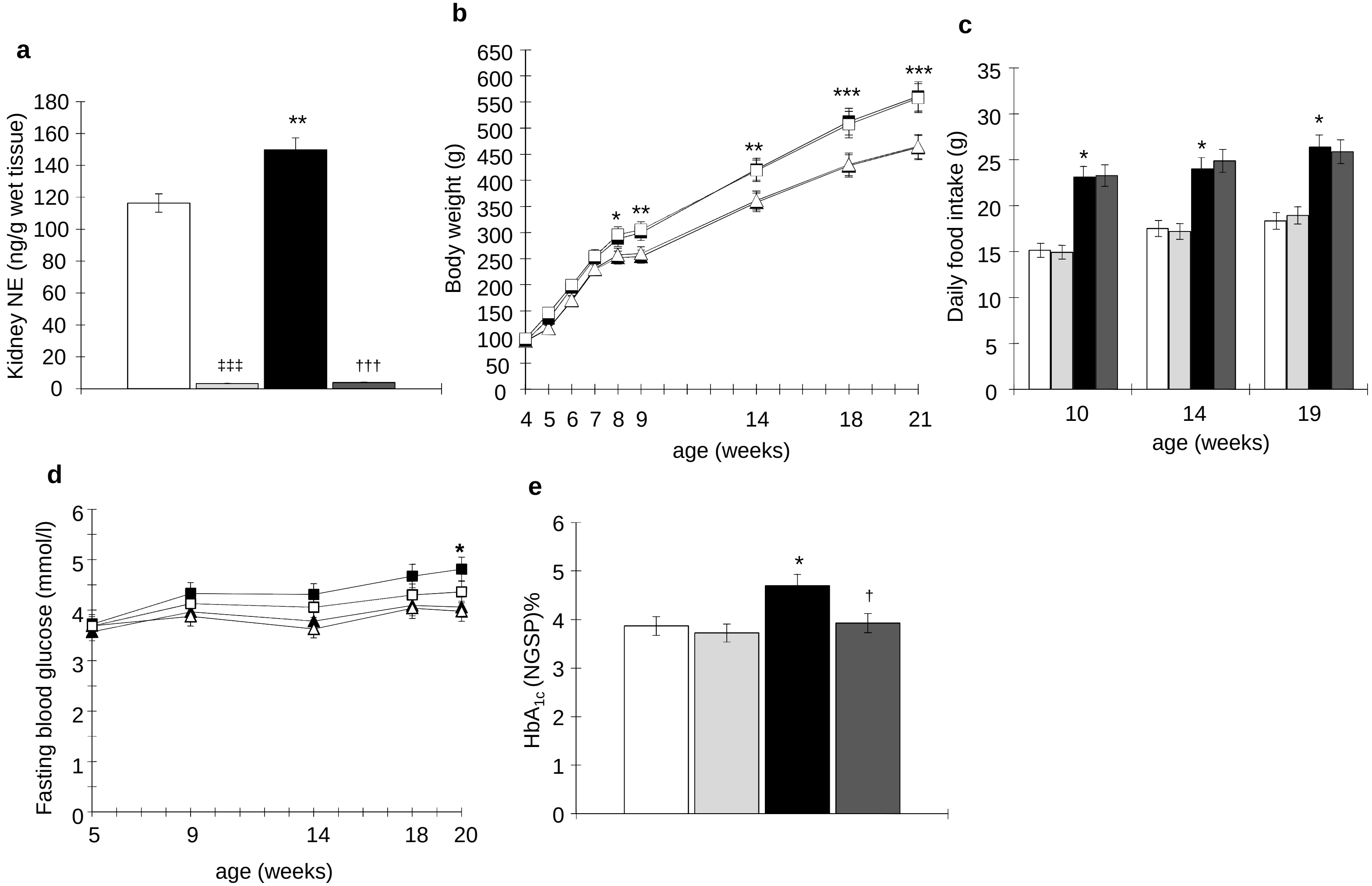

a
Kidney NE (ng/g wet tissue)
0
20
40
60
80
100
120
140
160
180
**
‡‡‡
†††
b
Body weight (g)
0
50
100
150
200
250
300
350
400
450
500
550
600
650
*
**
**
***
***
4 5 6 7 8 9 14 18 21
age (weeks)
c
Daily food intake (g)
0
5
10
15
20
25
30
35
*
*
*
10 14 19
age (weeks)
d
Fasting blood glucose (mmol/l)
0
1
2
3
4
5
6
*
5 9 14 18 20
age (weeks)
e
HbA1c (NGSP)%
0
1
2
3
4
5
6
*
†

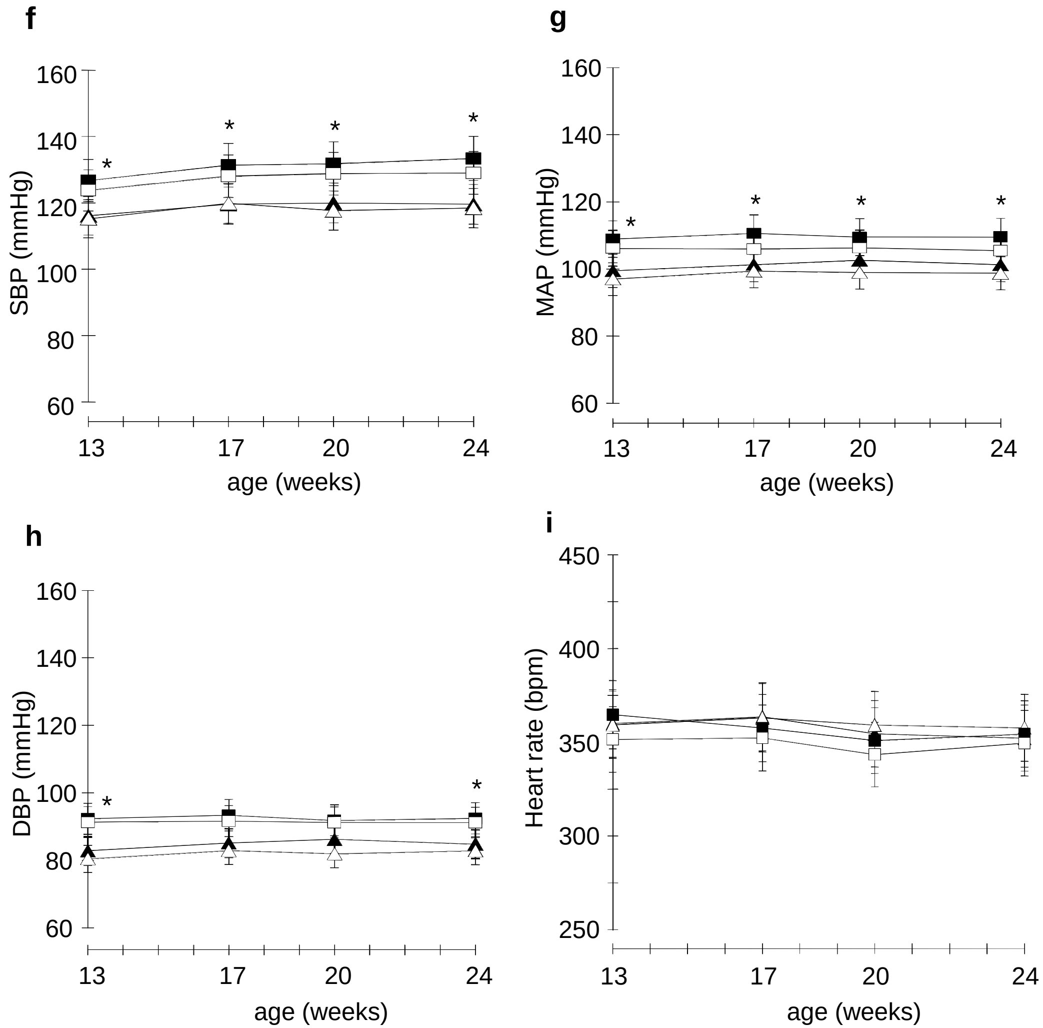
f
160
140
120
100
80
60
SBP (mmHg)
*
*
*
*
13
17
20
24
age (weeks)
g
160
140
120
100
80
60
MAP (mmHg)
*
*
*
*
13
17
20
24
age (weeks)
h
160
140
120
100
80
60
DBP (mmHg)
*
*
13
17
20
24
age (weeks)
i
450
400
350
300
250
Heart rate (bpm)
13
17
20
24
age (weeks)

ESM Fig. 1. Kidney tissue norepinephrine (NE), body weight changes and daily food intake, blood glucose, HbA_{1c} and blood pressure profiles in the pre-diabetic stage. **a,** OLETF rats show significantly higher kidney tissue NE levels compared with age matched LETO rats. Kidney tissue NE levels in OLETF rats subjected to renal sympathetic denervation (RDX) are almost undetectable (< 5 ng/g wet renal tissue). **b**, OLETF rats show increased body weight compared with LETO rats during the experimental period. RDX does not affect body weight, either in LETO or OLETF rats. **c**, OLETF rats show higher daily average food intake compared with LETO rats, which is unaffected by RDX. **d**, OLETF rats have elevated fasting blood glucose and HbA_{1c} (To convert values for HbA_{1c} in DCCT % into mmol/mol, subtract 2.15 and multiply by 10.929.) (**e**) levels compared with LETO rats, which were lowered by RDX (at 20 weeks of age). **f-h,** OLETF rats show increased systolic blood pressure (SBP) (**f**), mean arterial blood pressure (MAP) (**g**), and diastolic blood pressure (DBP) (**h**) compared with LETO rats. RDX does not significantly lower SBP, MAP or DBP. **i,** Heart rate is not significantly different between the groups. $^{*}p < 0.05$, $^{**}p < 0.01$ LETO *vs.* OLETF; $^{\dagger}p < 0.05$, $^{\dagger\dagger\dagger}P < 0.005$ OLETF *vs.* OLETF+RDX; $^{\ddagger}p < 0.05$ LETO *vs.* LETO+RDX. White bars, black triangles represent LETO group. Black bars, black squires represent OLETF group. Light grey bar, white triangles represent LETO+RDX. Dark grey bar, white squires represent OLETF+RDX.
