# Supplementary material for: Role of the renal sympathetic nerve in renal glucose metabolism during the development of type 2 diabetes in rats
Source: Diabetologia. 2015 Oct 8;58(12):2885–98. doi: 10.1007/s00125-015-3771-9 (PMC4630257; doi:10.1007/s00125-015-3771-9)
Supplement: Supplementary file 4 — (PDF 139 kb) [file 125_2015_3771_MOESM4_ESM.pdf]

ESM Fig. 2.

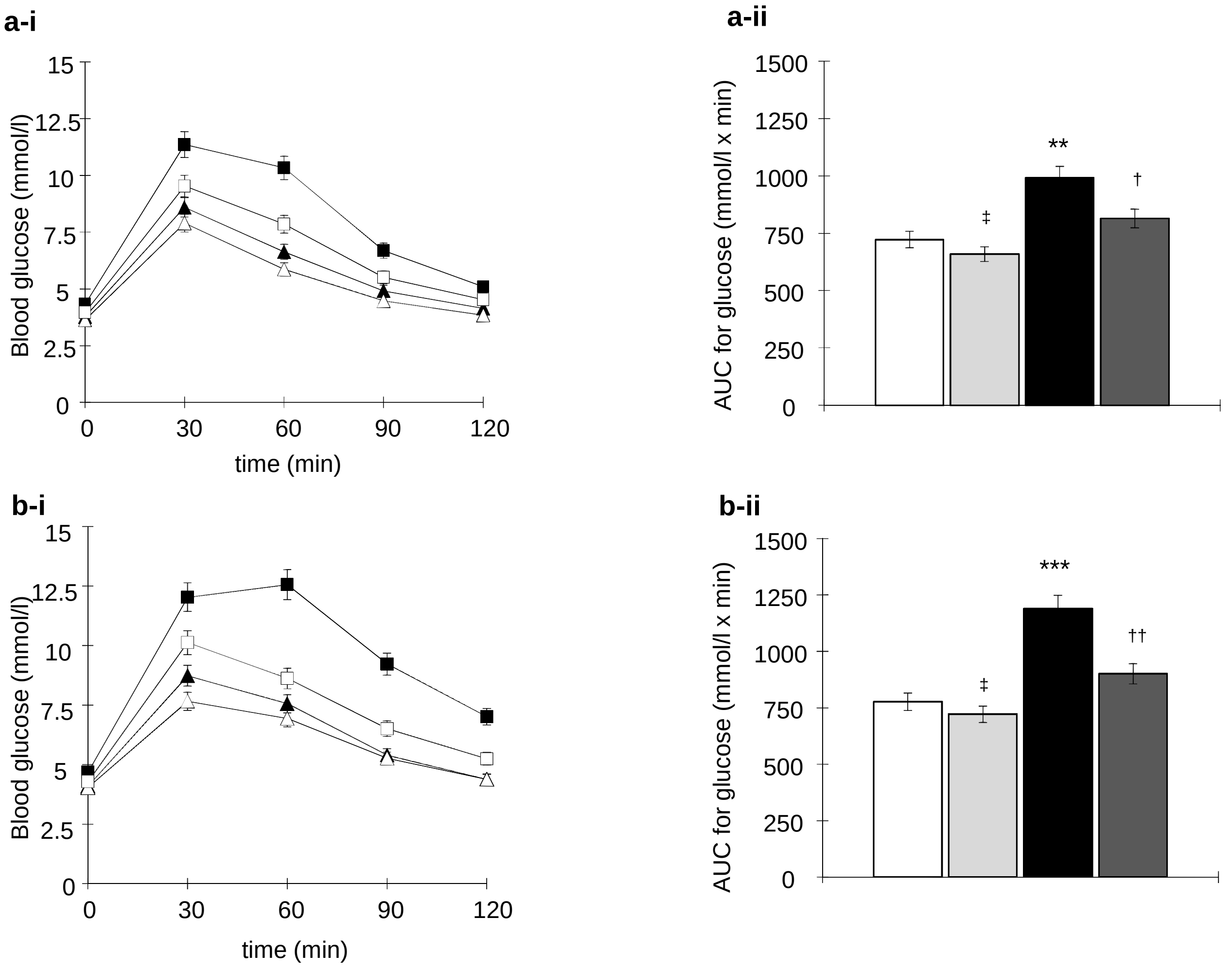
a-i
Blood glucose (mmol/l)
15
12.5
10
7.5
5
2.5
0
0
30
60
90
120
time (min)
a-ii
AUC for glucose (mmol/l x min)
1500
1250
1000
750
500
250
0
**
‡
†
b-i
Blood glucose (mmol/l)
15
12.5
10
7.5
5
2.5
0
0
30
60
90
120
time (min)
b-ii
AUC for glucose (mmol/l x min)
1500
1250
1000
750
500
250
0
***
‡
††

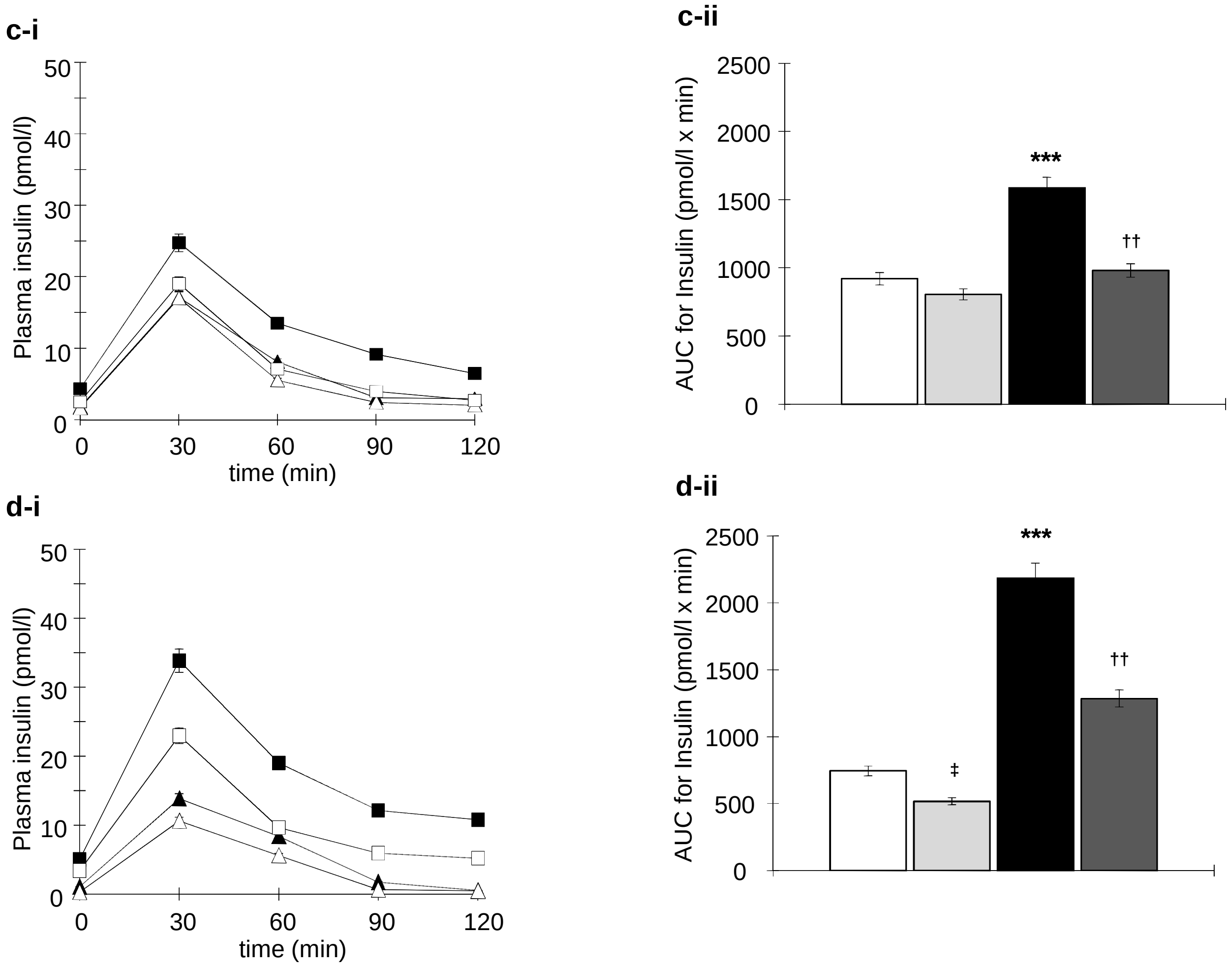
c-i
Plasma insulin (pmol/l)
time (min)
c-ii
AUC for Insulin (pmol/l x min)
***
††
d-i
Plasma insulin (pmol/l)
time (min)
d-ii
AUC for Insulin (pmol/l x min)
***
††
‡

ESM Fig. 2. Blood glucose and insulin levels, and their respective area under the curve (AUC), during the oral glucose tolerance test (OGTT). **a, b,** Blood glucose levels and their respective AUC during the OGTT at 14 (**a-i, a-ii**) and 20 (**b-i,b-ii**) weeks of age (8 and 14 weeks after RDX, respectively). Compared with LETO rats the OLETF rats showed higher glucose levels and associated AUC after oral administration of glucose, which were attenuated by RDX. **c, d,** Plasma insulin levels and associated AUC during OGTT at 14 (**c-i, c-ii**) and 20 (**d-i, d-ii**) weeks of age (8 and 14 weeks after RDX, respectively). OLETF rats have substantially greater plasma insulin levels and associated AUC after the oral administration of glucose compared with LETO rats, which was suppressed by RDX. $^{**}p < 0.01$, $^{***}p < 0.005$ LETO *vs.* OLETF; $^{\dagger}p < 0.05$, $^{\dagger\dagger}p < 0.01$ OLETF *vs.* OLETF+RDX; $^{\ddagger}p < 0.05$ LETO *vs.* LETO+RDX. White bars, black triangles represent LETO group. Black bars, black squires represent OLETF group. Light grey bar, white triangles represent LETO+RDX. Dark grey bar, white squires represent OLETF+RDX.
