# Supplementary material for: Role of the renal sympathetic nerve in renal glucose metabolism during the development of type 2 diabetes in rats
Source: Diabetologia. 2015 Oct 8;58(12):2885–98. doi: 10.1007/s00125-015-3771-9 (PMC4630257; doi:10.1007/s00125-015-3771-9)
Supplement: Supplementary file 5 — (PDF 24 kb) [file 125_2015_3771_MOESM5_ESM.pdf]

ESM Fig. 3.

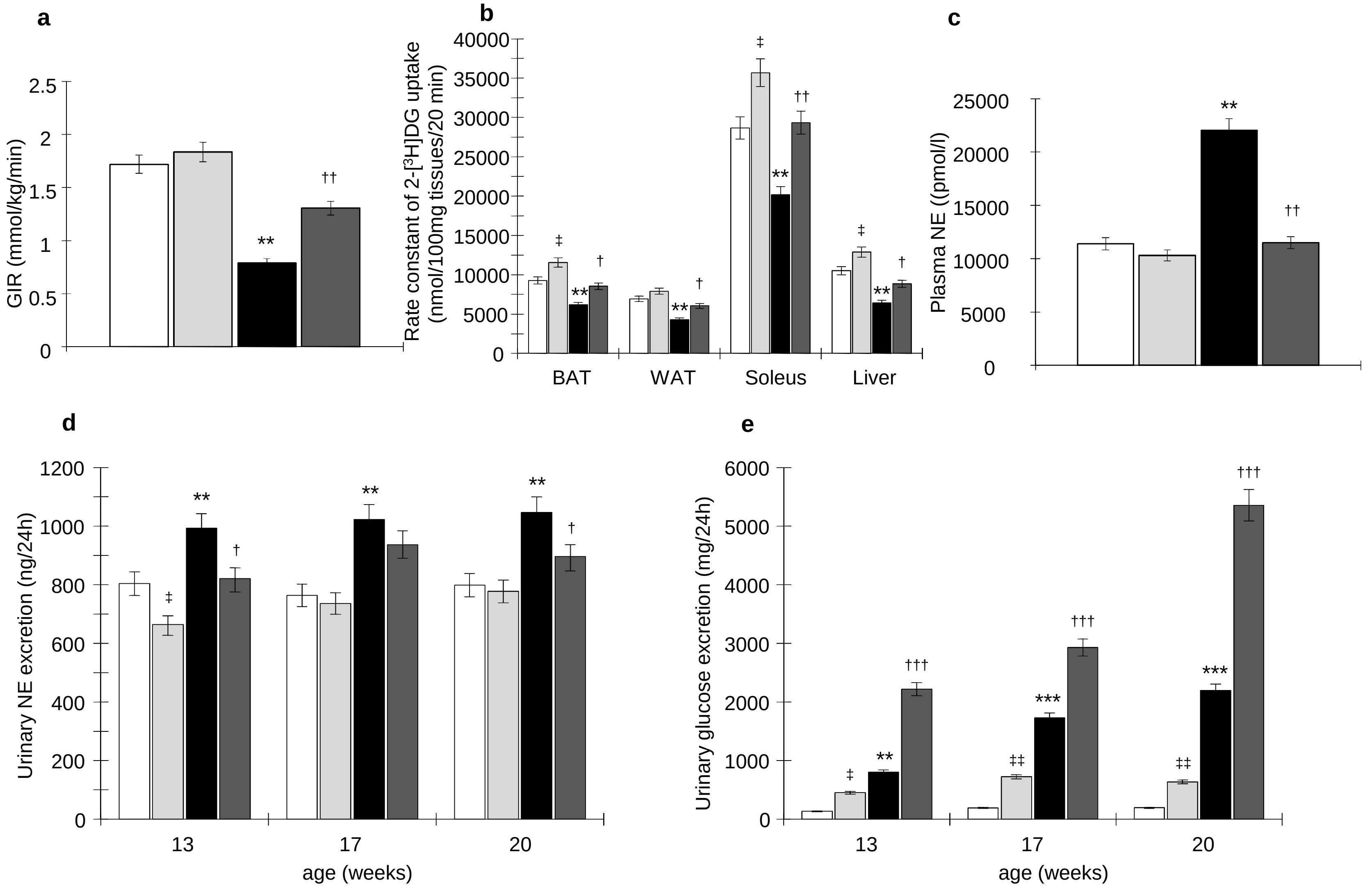
a
GIR (mmol/kg/min)
2.5
2
1.5
1
0.5
0
**
††
b
Rate constant of 2-[3H]DG uptake (nmol/100mg tissues/20 min)
40000
35000
30000
25000
20000
15000
10000
5000
0
BAT
WAT
Soleus
Liver
c
Plasma NE ((pmol/l)
25000
20000
15000
10000
5000
0
d
Urinary NE excretion (ng/24h)
1200
1000
800
600
400
200
0
13
17
20
age (weeks)
e
Urinary glucose excretion (mg/24h)
6000
5000
4000
3000
2000
1000
0
13
17
20
age (weeks)

ESM Fig. 3. Hyperinsulinemic-euglycemic clamp study, *in vivo* glucose uptake, plasma NE content, urine NE and glucose excretion in the pre-diabetic stage. **a**, OLETF rats show a markedly lowered glucose infusion rate (GIR) during the hyperinsulinemic-euglycemic clamp study compared with LETO rats, which was increased by RDX in OLETF rats. **b**, During rate constant of net tissue uptake of 2-[^{3}H]Deoxy-D-Glucose measurement, OLETF rats showed decreased glucose uptake in brown adipose tissues (BAT), white adipose tissues (WAT), soleus muscles and liver tissues compared with LETO rats. RDX increased glucose uptake by these tissues. **c, d**, OLETF rats show elevated plasma NE levels (**c**) and increased excretion of urine NE (**d**) compared with LETO rats. RDX suppresses plasma NE contents and attenuates urine NE excretion. **e**, OLETF rats showed increased urine glucose excretion compared LETO rats. Renal denervated OLETF rats show markedly increased urine glucose excretion compared with non-denervated OLETF rats. $^{**}p < 0.01$, $^{***}p < 0.005$ LETO *vs.* OLETF; $^{\dagger}p < 0.05$, $^{\dagger\dagger}P < 0.01$, $^{\dagger\dagger\dagger}p < 0.005$ OLETF *vs.* OLETF+RDX; $^{\ddagger}p < 0.05$, $^{\ddagger\ddagger}p < 0.01$ *vs.* LETO+RDX. White bars represent LETO group. Black bars represent OLETF group. Light grey bar represent LETO+RDX. Dark grey bar represent OLETF+RDX.
