# Supplementary material for: Role of the renal sympathetic nerve in renal glucose metabolism during the development of type 2 diabetes in rats
Source: Diabetologia. 2015 Oct 8;58(12):2885–98. doi: 10.1007/s00125-015-3771-9 (PMC4630257; doi:10.1007/s00125-015-3771-9)
Supplement: Supplementary file 6 — (PDF 208 kb) [file 125_2015_3771_MOESM6_ESM.pdf]

ESM Fig. 4.

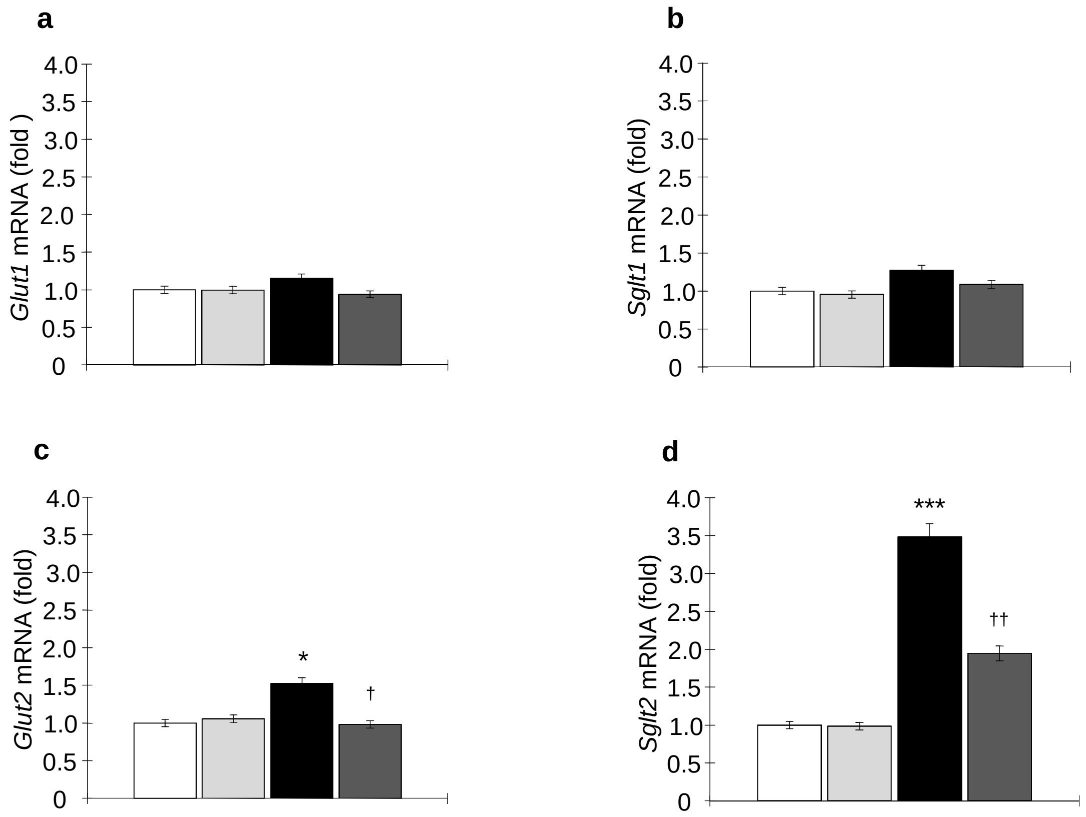
a
4.0
3.5
3.0
2.5
2.0
1.5
1.0
0.5
0
Glut1 mRNA (fold)
b
Sglt1 mRNA (fold)
c
Glut2 mRNA (fold)
*
†
d
Sglt2 mRNA (fold)
***
††

e

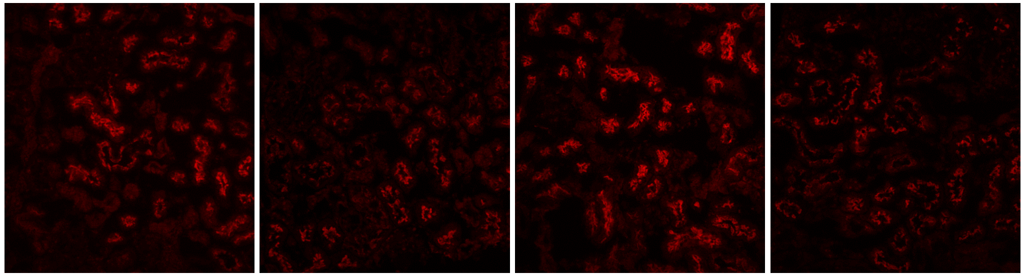

ESM Fig. 4. Expression of glucose transporters in the kidney. **a, b,** Renal cortical tissue *Glut1* (**a**) and *Sglt1* (**b**) mRNA levels were similar between treatment groups. However, *Glut2* (**c**) mRNA expression shows significant upregulation in OLETF rats compared with LETO rats. Furthermore, *Sglt2* (**d**) mRNA levels were markedly upregulated in renal cortical tissue of OLETF rats compared with LETO rats. The mRNA upregulation in OLETF rats was suppressed by RDX. Real-time reverse transcription polymerase chain reaction (RT-PCR) data are expressed as fold-changes compared with LETO rats after normalization for the expression of *β*-actin. **e**, Immunofluorescence micrographs demonstrating staining with an anti-*SGLT2* antibody (red fluorescence) (original magnification, ×200). The staining intensity is weaker in OLETF+RDX than OLETF rats. $^{*}p < 0.05$, $^{***}p < 0.005$ LETO *vs.* OLETF; $^{\dagger}p < 0.05$, $^{\dagger\dagger}p < 0.01$ OLETF *vs.* OLETF+RDX. White bars represent LETO group. Black bars represent OLETF group. Light grey bar represent LETO+RDX. Dark grey bar represent OLETF+RDX.
