# Supplementary material for: Role of the renal sympathetic nerve in renal glucose metabolism during the development of type 2 diabetes in rats
Source: Diabetologia. 2015 Oct 8;58(12):2885–98. doi: 10.1007/s00125-015-3771-9 (PMC4630257; doi:10.1007/s00125-015-3771-9)
Supplement: Supplementary file 7 — (PDF 381 kb) [file 125_2015_3771_MOESM7_ESM.pdf]

ESM Fig. 5.

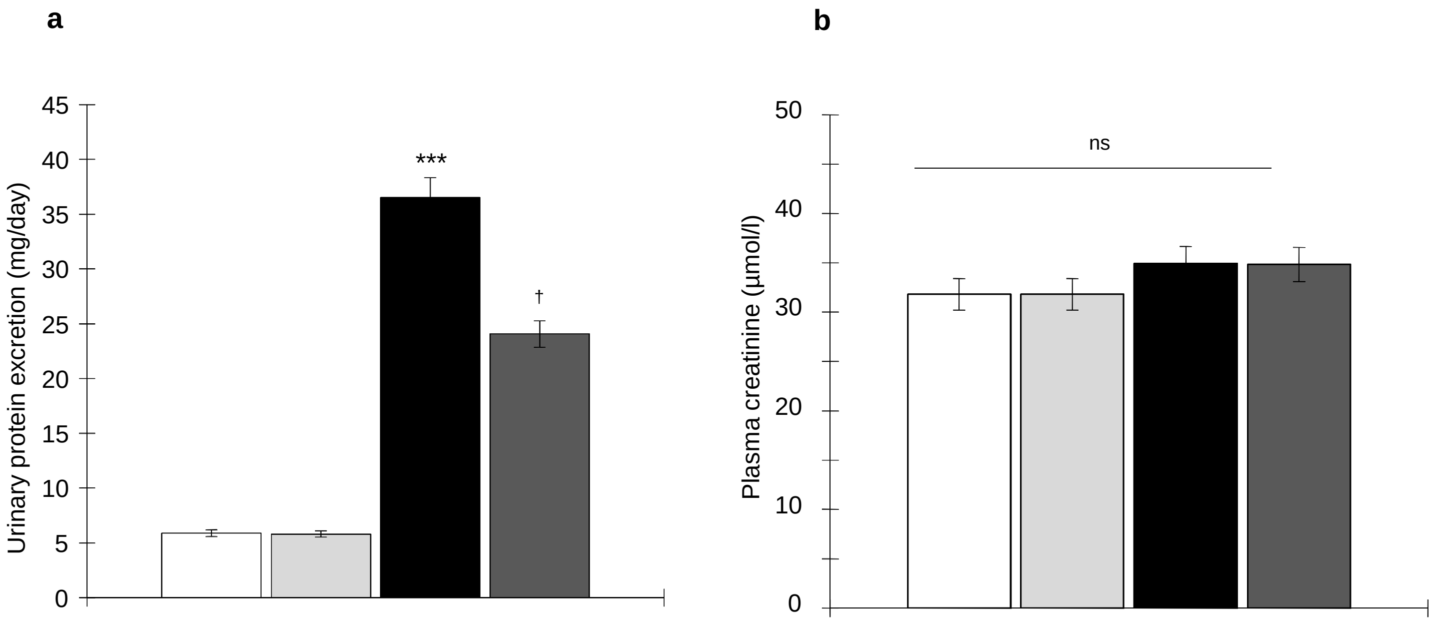

a
b
Urinary protein excretion (mg/day)
0
5
10
15
20
25
30
35
40
45
***
†
Plasma creatinine (µmol/l)
0
10
20
30
40
50
ns

c

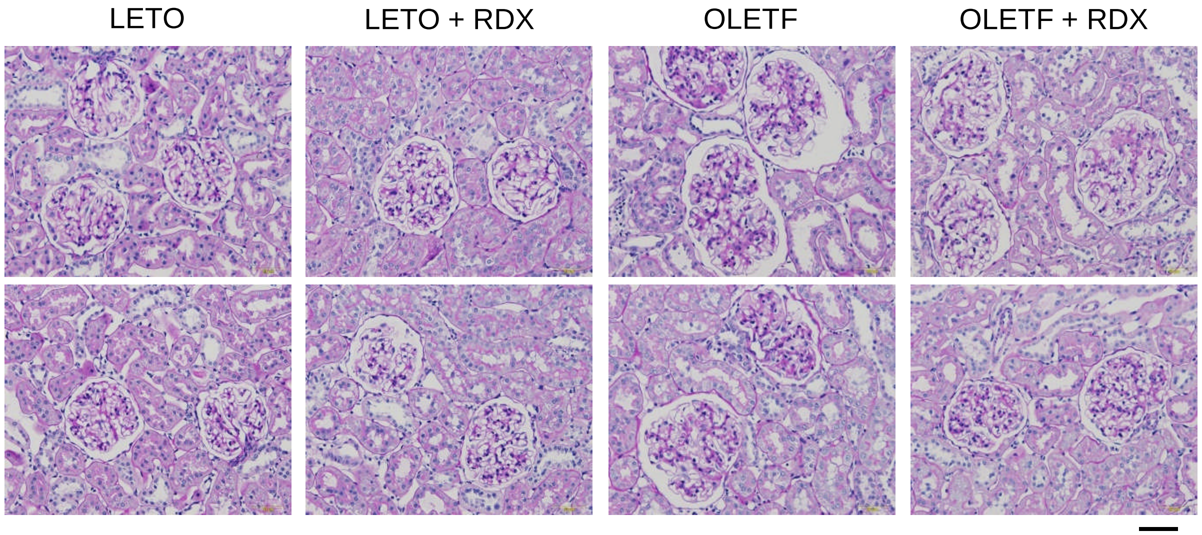
LETO
LETO + RDX
OLETF
OLETF + RDX

d

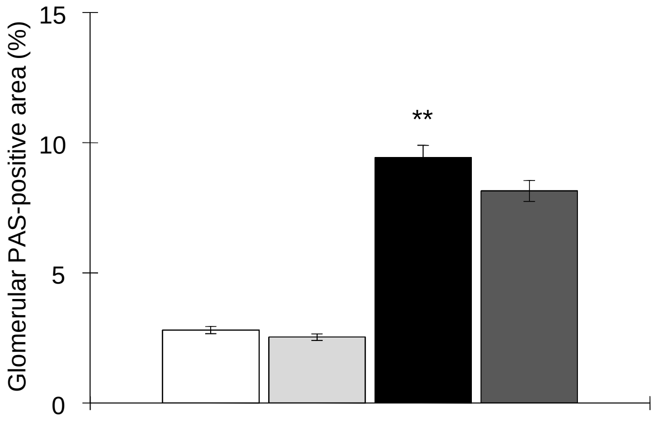
Glomerular PAS-positive area (%)
15
10
5
0
**

ESM Fig. 5. Proteinuria and plasma creatinine levels were determined, and renal structural changes evaluated by examining periodic acid-Schiff (PAS) staining. **a**, OLETF rats show increased urine protein excretion compared with LETO rats which is reduced by RDX. However, plasma creatinine concentration was not significantly altered between groups (**b**). **c**, Representative micrographs of PAS-stained renal sections (scale bar shows the values), and the PAS-positive area within the total glomerular area (**d**). OLETF rats show increased PAS-positive area and glomerular hypertrophy compared with LETO rats. RDX does not significantly affect these parameters. $^{**}p < 0.01$, $^{***}p < 0.005$ LETO *vs.* OLETF; $^{\dagger}p < 0.05$ OLETF *vs.* OLETF+RDX. Scale bar represent 50 µm. White bars represent LETO group. Black bars represent OLETF group. Light grey bar represent LETO+RDX. Dark grey bar represent OLETF+RDX.
