# Supplementary material for: Role of the renal sympathetic nerve in renal glucose metabolism during the development of type 2 diabetes in rats
Source: Diabetologia. 2015 Oct 8;58(12):2885–98. doi: 10.1007/s00125-015-3771-9 (PMC4630257; doi:10.1007/s00125-015-3771-9)
Supplement: Supplementary file 8 — (PDF 36 kb) [file 125_2015_3771_MOESM8_ESM.pdf]

ESM Fig. 6.

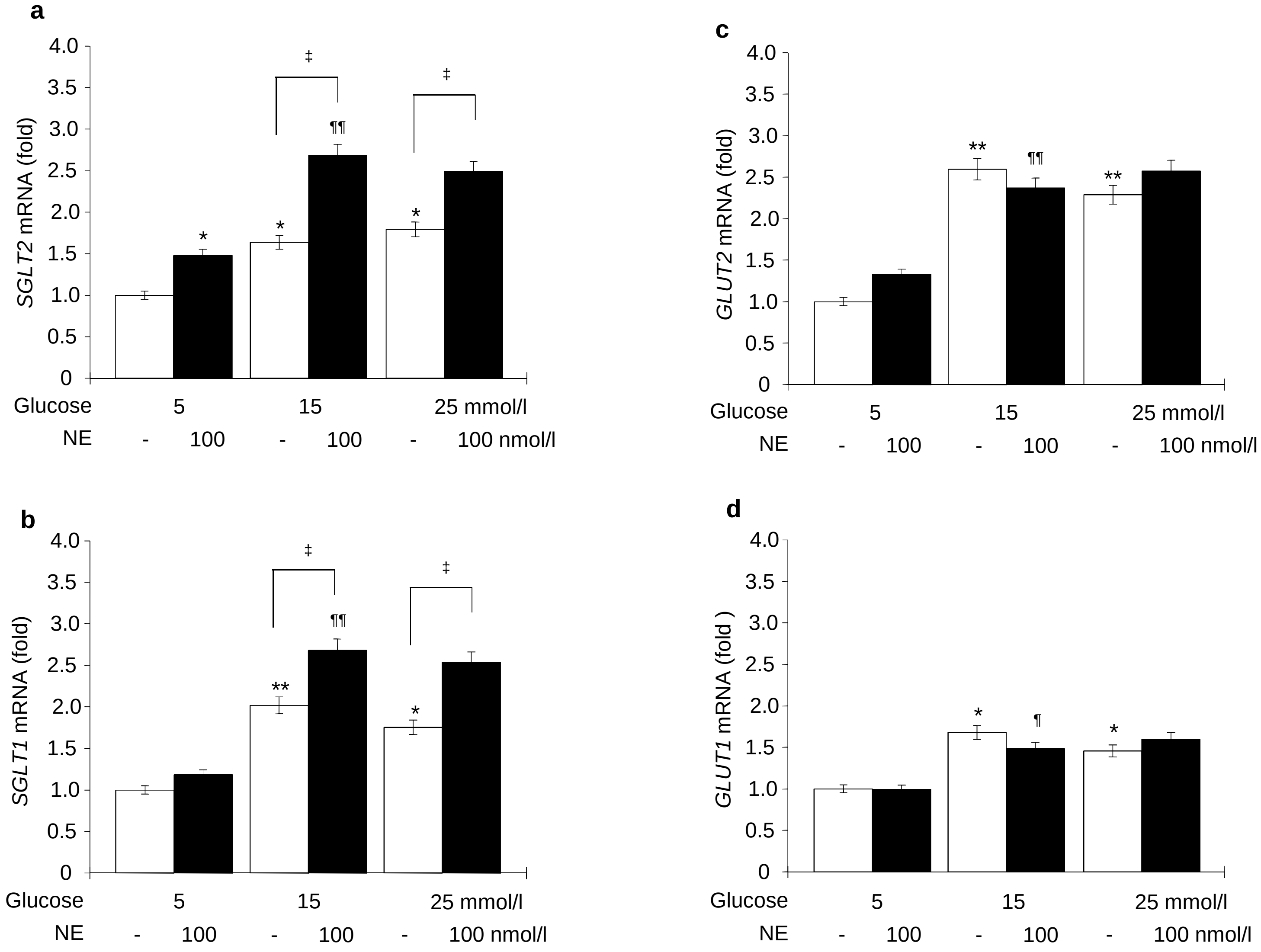

a
4.0
3.5
3.0
2.5
2.0
1.5
1.0
0.5
0
SGLT2 mRNA (fold)
‡
‡
¶¶
*
*
*
Glucose
5
15
25 mmol/l
NE
-
100
-
100
-
100 nmol/l
b
SGLT1 mRNA (fold)
‡
‡
¶¶
**
*
c
GLUT2 mRNA (fold)
**
¶¶
**
d
GLUT1 mRNA (fold)
*
¶
*

ESM Fig. 6. Effects of high glucose and norepinephrine (NE) on glucose transporter gene expression in human kidney proximal tubule epithelial cells (HK2). **a**, Treatment with high glucose for 24 hours upregulated *SGLT2* mRNA expression, which was further enhanced by exposure to NE. **b**, *GLUT2* mRNA expression was also upregulated by high glucose treatment, however the addition of NE for 24 h did not significantly alter such values. **c**, Treatment with high glucose for 24 h upregulated *SGLT1* mRNA expression, which was further enhanced by exposure to NE. **d**, *GLUT1* mRNA expression tended to be upregulated by high glucose treatment. However, the addition of NE for 24 h did not significantly alter these values. Data are expressed as the relative difference in expression compared with 5 mmol/l glucose after normalization for *β*-actin expression. $^{*}p < 0.05$, $^{**}p < 0.01$ *vs.* 5 mmol/l glucose; $^{¶}p < 0.05$, $^{¶¶}p < 0.01$ *vs.* 5 mmol/l glucose+NE.$^{‡}p < 0.05$.
