# Supplementary material for: Role of the renal sympathetic nerve in renal glucose metabolism during the development of type 2 diabetes in rats
Source: Diabetologia. 2015 Oct 8;58(12):2885–98. doi: 10.1007/s00125-015-3771-9 (PMC4630257; doi:10.1007/s00125-015-3771-9)
Supplement: Supplementary file 9 — (PDF 24 kb) [file 125_2015_3771_MOESM9_ESM.pdf]

ESM Table 1. The rat oligonucleotide primer sequences for real-time RT-PCR (*in vivo* studies).

Name		Primer Sequences
Glut1	sense	CTGTTGGCCTTTGTGTCTGC
	antisense	GTCAGGCCACAGTACACTCC
Glut2	sense	ACCAGCACATACGACACCAG
	antisense	ACCATTCCGCCTACTGCAAA
Glut4	sense	CCTTTGCACACCACTTCCGA
	antisense	AGAGCCGATCTGCTGGAAAC
Sglt1	sense	TCTTCGCTATCAGCGTCGTC
	antisense	TGCGCTCTTCTGTGCTGTTA
Sglt2	sense	GACATTCTGGTCATTGCCGC
	antisense	CTGCCAAGAAGTAGCCACCA
β-actin	sense	ACAACCTTCTTGCAGCTCCTCCG
	antisense	ACGAGCGCAGCGATATCGTC

Glut1, glucose transporter 1; *Glut2*, glucose transporter 2; *Glut4*, glucose transporter 4; *Sglt1*, sodium dependent glucose transporter 1; *Sglt2*, sodium dependent glucose transporter 2.
