# Supplementary material for: Role of the renal sympathetic nerve in renal glucose metabolism during the development of type 2 diabetes in rats
Source: Diabetologia. 2015 Oct 8;58(12):2885–98. doi: 10.1007/s00125-015-3771-9 (PMC4630257; doi:10.1007/s00125-015-3771-9)
Supplement: Supplementary file 10 — (PDF 24 kb) [file 125_2015_3771_MOESM10_ESM.pdf]

ESM Table 2. The human oligonucleotide primer sequences for real-time RT-PCR (*in vitro* studies).

Name		Primer Sequences
GLUT1	sense	GCCTGGATCTCCCCACTCTA
	antisense	CAGTGCTCCCAACTGGTCTC
GLUT2	sense	TCCAGCTACCGACAGCCTAT
	antisense	GCTGATACCAGCCGTCTGAA
SGLT1	sense	AGCAGACTTGTGTGGAAGCA
	antisense	GGGAGGCTCCAATCTCATGT
SGLT2	sense	CTGGTCATTGGCGTTGGCTT
	antisense	ATGTTGCTGGCGAAGAGAGAG
β-actin	sense	CACAGAGCCTCGCCTTTGCCGATC
	antisense	ACGAGCGCGGCGATATCATCATC

GLUT1, glucose transporter 1; *GLUT2*, glucose transporter 2; *SGLT1*, sodium dependent glucose transporter 1; *SGLT2*, sodium dependent glucose transporter 2.
