# Supplementary material for: Role of the renal sympathetic nerve in renal glucose metabolism during the development of type 2 diabetes in rats
Source: Diabetologia. 2015 Oct 8;58(12):2885–98. doi: 10.1007/s00125-015-3771-9 (PMC4630257; doi:10.1007/s00125-015-3771-9)
Supplement: Supplementary file 11 — (PDF 8 kb) [file 125_2015_3771_MOESM11_ESM.pdf]

ESM Table 3. the expression of *Glut4* in skeletal muscle at the diabetic stage (protocol-1) and at the pre-diabetic stage (protocol-2).

	Diabetic stage (at 46 weeks of age) (fold changes)	Pre-diabetic stage (at 21 weeks of age) (fold changes)
LETO (n = 8)	1.00±0.40	1.00±0.3
LETO + RDX (n = 8)	1.01±0.27	1.03±0.25
OLETF(n = 8)	0.62±0.11††	0.70±0.18†
OLETF + RDX (n = 8)	0.95±0.04‡‡	0.91±0.09‡

Data are means±SEM. $^{†}P < 0.05$, $^{††}P < 0.01$ LETO *vs.* OLETF; $^{‡}P < 0.05$, $^{‡‡}P < 0.01$ OLETF *vs.* OLETF + RDX. *Glut4*, glucose transporter 4.
